# Retinoic acid receptor α as a novel contributor to adrenal cortex structure and function through interactions with Wnt and Vegfa signalling

**DOI:** 10.1038/s41598-019-50988-2

**Published:** 2019-10-11

**Authors:** Rami M. El Zein, Audrey H. Soria, Jose Felipe Golib Dzib, Amanda J. Rickard, Fabio L. Fernandes-Rosa, Benoit Samson-Couterie, Isabelle Giscos-Douriez, Angélique Rocha, Marko Poglitsch, Celso E. Gomez-Sanchez, Laurence Amar, Norbert B. Ghyselinck, Arndt Benecke, Maria-Christina Zennaro, Sheerazed Boulkroun

**Affiliations:** 1 INSERM, UMRS_970, Paris Cardiovascular Research Center, Paris, France; 2 Université Paris Descartes, Sorbonne Paris Cité, Paris, France; 3 Centre National de la Recherche Scientifique (CNRS), Institut des Hautes Etudes Scientifiques, Bures sur Yvette, France; 4 Attoquant Diagnostics, Vienna, Austria; 5 Division of Endocrinology, G.V. (Sonny) Montgomery VA Medical Center and University of Mississippi Medical Center, Jackson, MS 39216 USA; 6 Assistance Publique-Hôpitaux de Paris, Hôpital Européen Georges Pompidou, Unité Hypertension artérielle, Paris, France; 7 Institut de Génétique et de Biologie Moléculaire et Cellulaire (IGBMC), Département de Génétique Fonctionnelle et Cancer; CNRS UMR7104, Illkirch, France; 8 INSERM, U1258 Illkirch, France; 9 Université de Strasbourg (UNISTRA), Illkirch Cedex, France; 10 Present Address: CNRS UMR8246, NPS, Sorbone University, Paris, France; 11 Assistance Publique-Hôpitaux de Paris, Hôpital Européen Georges Pompidou, Service de Génétique, Paris, France

**Keywords:** Cardiovascular diseases, Cell signalling, Nuclear receptors

## Abstract

Primary aldosteronism (PA) is the most frequent form of secondary arterial hypertension. Mutations in different genes increase aldosterone production in PA, but additional mechanisms may contribute to increased cell proliferation and aldosterone producing adenoma (APA) development. We performed transcriptome analysis in APA and identified retinoic acid receptor alpha (RARα) signaling as a central molecular network involved in nodule formation. To understand how RARα modulates adrenal structure and function, we explored the adrenal phenotype of male and female Rarα knockout mice. Inactivation of Rarα in mice led to significant structural disorganization of the adrenal cortex in both sexes, with increased adrenal cortex size in female mice and increased cell proliferation in males. Abnormalities of vessel architecture and extracellular matrix were due to decreased Vegfa expression and modifications in extracellular matrix components. On the molecular level, *Rarα* inactivation leads to inhibition of non-canonical Wnt signaling, without affecting the canonical Wnt pathway nor PKA signaling. Our study suggests that Rarα contributes to the maintenance of normal adrenal cortex structure and cell proliferation, by modulating Wnt signaling. Dysregulation of this interaction may contribute to abnormal cell proliferation, creating a propitious environment for the emergence of specific driver mutations in PA.

## Introduction

Primary aldosteronism (PA) is the most common and curable form of secondary arterial hypertension, with prevalence estimations of up to 10% of cases in referred hypertensive patients, 4% of patients in primary care^1,2^ and 20% of patients with resistant hypertension^3,4^. Rapid diagnosis and treatment are important to prevent severe cardiovascular consequences of long term aldosterone exposure, which are independent of blood pressure levels and are due to deleterious cardiovascular effects of the hormone^5,6^. Indeed, patients with PA have an increased risk of developing stroke, coronary artery disease, atrial fibrillation, left ventricular hypertrophy, and heart failure^2,5,7–9^. In the majority of cases, PA is due to an aldosterone producing adenoma (APA) or bilateral adrenal hyperplasia (BAH)^10^. Patients with PA present hypertension, a high aldosterone to renin ratio and variable hypokalemia^11^. Over recent years, whole exome sequencing (WES) unraveled different somatic mutations in genes encoding ion channels (*KCNJ5* and *CACNA1D*) and ATPases (*ATP1A1* and *ATP2B3*)^12–15^ in about 50% of APA^16^. Recently, use of aldosterone synthase immunohistochemistry-guided next-generation sequencing identified somatic mutations in up to 88% of APA^17^. In physiological conditions, these channels and pumps play an important role in regulating intracellular ion homeostasis as well as maintaining cell membrane potential. When mutated, they induce cell membrane depolarization leading to opening of voltage-gated calcium channels, or directly affect intracellular calcium concentrations. This ultimately leads to activation of calcium signaling, the main trigger of aldosterone production. Although the link between mutations and aldosterone production has been clearly established, their role in promoting abnormal cell proliferation and/or APA development is not well understood. Activating mutations in the *CTNNB1* gene (encoding β-catenin) were also identified in 2–5% of APA^18,19^, and the Wnt/β-catenin signaling pathway has been shown to be constitutively active in ~70% of APA^20,21^. This signaling pathway plays an important role in the development of the adrenal cortex and in aldosterone biosynthesis^22^. Recent studies have suggested a two-hit mechanism of APA development, whereby a first hit induces adrenal cortex remodeling and/or increases nodule formation and a second hit, involving mutations in APA driver genes, specifies the hormonal secretory pattern^23,24^.

In mice, the adrenal cortex is composed of two distinct functional zones, the zona glomerulosa (ZG) and the zona fasciculata (ZF), with different functions. The ZG is located under the capsule and produces mineralocorticoids that play a major role in the regulation of blood pressure by regulating sodium and potassium homeostasis. The ZF produces glucocorticoid hormones that are involved in stress response and energy homeostasis. Adrenal cortex undergoes continual renewal, with stem/progenitor cells that first differentiate into ZG cells and then migrate centripetally acquiring ZF cells characteristics^25^. Different studies report sexual dimorphism in mouse adrenal cortex, with adrenals being larger in females than in males and plasma ACTH, corticosterone and aldosterone levels being higher^26^. At the transcriptome level, a core sexually dimorphic expression program has been identified^26^. Moreover, sexual dimorphism in the adrenal cortex pathophysiology has been reported in several genetically modified mouse models^21,27–29^. Interestingly, adrenal cortex renewal has been recently shown to be 3-fold faster in females than in males, highlighting the role of sex hormones in this process^30^.

Here we have performed a large-scale study integrating transcriptome, histological and immunohistological analyses with clinical and biological information of patients with APA to better understand the mechanisms involved in increased cell proliferation in BAH and APA development and to identify specific signaling pathways responsible for abnormal cell proliferation and nodule formation. We identified retinoic acid receptor (RAR) α signaling as a central molecular network involved in nodule formation. Analysis of the adrenal phenotype of mice lacking *Rarα* revealed structural and functional disorganization of the adrenal cortex at 12 weeks of age, which was associated with modifications of the extracellular matrix and vessel architecture in both male and female mice. This was accompanied by increased adrenal cortex weight in female, and increased cell proliferation in male mice. In males, morphological abnormalities were associated with alterations in non-canonical Wnt signaling as well as reduced expression of steroidogenic genes, without modifications in canonical Wnt signaling nor PKA signaling. Abnormalities of vessel architecture and extracellular matrix were due to decreased Vegfa expression and modifications in extracellular matrix components. With aging, adrenal cortex disorganization was still present, but without evidence of modification of Wnt signaling and Vegfa or steroidogenic gene expression. In female *Rarα*^*−/−*^ mice, morphological abnormalities of the adrenal cortex were also present at both ages. However, even at 12 weeks, no alterations in Wnt signaling and Vegfa expression were observed, suggesting that molecular abnormalities may have occurred at an earlier time point in adrenal cortex development. Our study suggests that Rarα contributes to the maintenance of normal adrenal cortex structure and cell proliferation, by modulating Wnt signaling. Dysregulation of this interaction may contribute to abnormal cell proliferation, creating a propitious environment for the emergence of specific driver mutations in PA.

## Results

### Decrease of retinoic acid receptor α expression is associated with nodulation in APA

**Figure 1:**
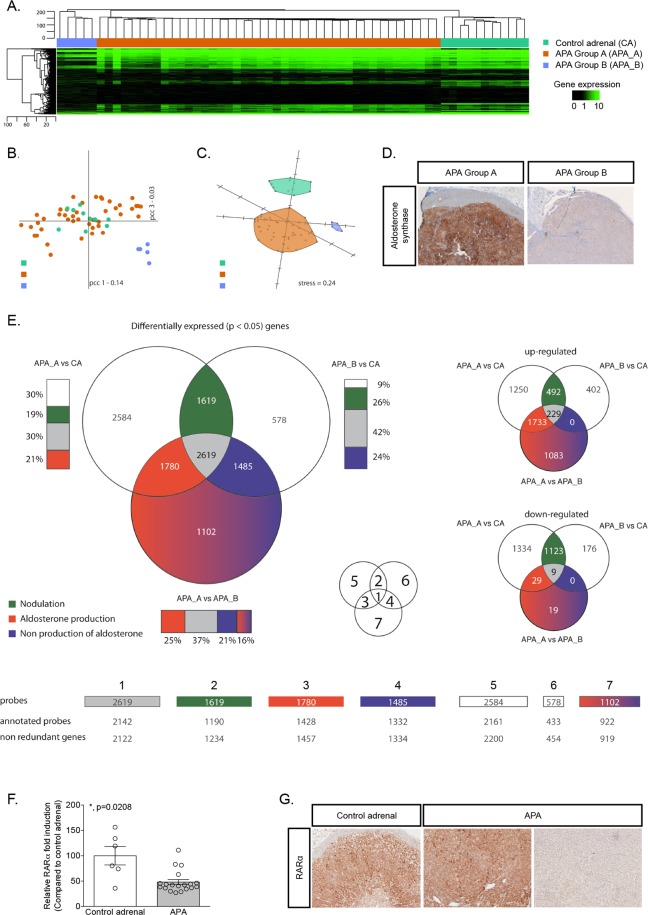
Transcriptome analysis reveals two distinct subgroups of APA. (**A**) Hierarchical clustering using Euclidean distance and complete linkage of 48 APA and 11 control adrenals. Heat-map of the whole gene expression is displayed using black for low expression and green for high expression. (**B**) Principal Component Analysis in the correlation space over the entire transcriptome. The first two principal components and their associated inertia are displayed. (**C**) Singular-value-decomposition-initialized multidimensional scaling under the correlation space over all genes^86,87^. Kruskal error is indicated as “stress”. (**D**) Aldosterone synthase immunohistochemistry performed on adrenals from APA_A and APA_B. (**E**) Three groups (APA_A, APA_B and CA) are pair-wise compared to obtain a list of statistically significantly expressed candidate genes for each of the three comparisons. The common and comparison-specific genes are classified in seven different groups some of which are associated to a biological process. Venn-diagrams are displayed for statistically significantly differentially expressed candidate genes. The smaller Venn diagrams on the right-hand side of the figure represent the overlap for up-regulated and down-regulated genes. Bars are displayed next to the represented sets to indicate the relative proportion of the overlapping regions to the corresponding list of candidate genes. Figures displayed are the number of genes that populate each of the selected sets. (**F**) Expression of *RARα* was investigated by RT-qPCR on mRNA extracted from 6 control adrenals and 19 APA. Values are presented as the mean ± SEM; p values were calculated using a two-sided Mann-Whitney test. *p < 0.05. (**G**) RARα immunohistochemistry performed on control adrenal and in APA.

We compared the transcriptome profile of 48 APA and 11 control adrenals. Unsupervised clustering revealed the presence of at least three different groups of samples (Fig. 1A). The first one being composed exclusively of control adrenals, whereas the 48 APA were subdivided into two groups of 43 and 5 samples defined as APA group A (APA_A) and APA group B (APA_B), respectively. The APA_B is spatially separated from the rest of the samples (Fig. 1B), and from this perspective control adrenals seem to be very similar to samples in APA_A. However, using the first three dimensions of a Principal Component Analysis (PCA), APA_A are clearly separated from control adrenals and from APA_B (Fig. 1C). By comparing clinical and biochemical characteristics of patients from APA_A and APA_B, we found no difference between groups except for the tumor size [15 mm (9–18) for APA_A versus 40 mm (32–52.5) for APA_B (Table 1)]. Interestingly, another major difference characterizing these two groups of tumors was the level of expression of CYP11B2 (aldosterone synthase) detected by immunohistochemistry, which was higher in APA_A compared to APA_B (Fig. 1D). The three groups were pairwise compared to obtain a list of statistically significantly differentially expressed genes for each of the three comparisons (Supplementary Tables 1–3). The common and comparison-specific genes were then classified into 7 different groups, some of which were associated to a specific biological process (Fig. 1E and Supplementary Fig. 1; Supplementary Tables 4–10). In particular, we made the hypothesis that genes commonly differentially expressed in APA_A (high aldosterone production) and APA_B (low aldosterone production) compared to control adrenals would be involved in nodulation (Fig. 1E, subgroup 2) rather than steroid biosynthesis. Within this group, the expression of retinoic acid receptor α (RARα) was significantly down regulated in APA (logQ[APA_A vs CA] -3.8852; logQ[APA_B vs CA] -3.0056). RT-qPCR performed on 6 control adrenals and 19 APA confirmed the down-regulation of *RARα* mRNA expression in APA (Fig. 1F). Immunohistochemistry showed that RARα was expressed throughout the adrenal cortex, and to a lesser extent in the medulla; its expression was heterogeneous in APA (Fig. 1G). Our transcriptome data also revealed that all the enzymes necessary for the biosynthesis of RA are expressed in control adrenal cortex as well as in APA (Table 2), supporting the possibility of RA biosynthesis and action in the adrenal gland. Modulation of RARα target genes was investigated by retrieving, from our transcriptome data, the expression of 511 genes described as RARα target genes and expressed in humans^31^ (Supplementary Table 11). Among them, 147 were found to be differentially expressed between APA_A and control adrenals (67 were up-regulated and 80 down-regulated) and 123 between APA_B and control adrenals (10 up-regulated and 113 down-regulated).

**Table 1:** Clinical and biochemical correlates of patients from group A and B.

	Group A	Group B	P value
Gender (male/female), %	51/49	40/60	1
Age at PA dg (ys)	39 (33, 44.5)	43 (36.5, 51)	0.29
Lowest plasma K (mmol/l)	3.2 (2.9, 3.5)	3.1 (3.0, 3.55)	0.78
Systolic BP (mmHg)	150 (135, 167)	154 (143, 175)	0.49
Diastolic BP (mmHg)	90 (82, 99)	95 (85, 102)	0.56
Treatment score (n)	2 (1, 3.5)	1 (0, 2)	0.07
Urinary aldosterone (nmol/24 h)	95 (76, 126)	115 (87.5, 153.5)	0.57
Plasma aldosterone (pmol/l)	845 (508, 1127)	1168 (633.5, 1797)	0.32
Plasma renin (mU/l)	2.2 (1.4, 4.3)	1.7 (1.2, 3.4)	0.42
ARR (pmol/mU)	271.8 (179.8, 602.5)	831.0 (385.5, 1152)	0.16
Adenoma size (mm)	14.5 (9.0, 18.0)	40 (32.0, 52.5)	<0.0001
Systolic BP at FU (mmHg)	131 (120, 149)	116 (112, 142)	0.31
Diastolic BP at FU (mmHg)	84 (80, 96)	73 (72, 94)	0.34
Treatment score at FU (n)	0 (0, 2)	0 (0, 2)	0.63
Plasma K at FU (mmol/l)	4.1 (3.8, 4.3)	4.0 (3.6, 4.2)	0.45
Plasma aldosterone at FU (pmol/l)	130 (85, 251)	74 (36, 200)	0.15
Plasma renin at FU (mU/l)	11.0 (6.8, 17.2)	9.0 (7.9, 101.3)	0.80
ARR at FU (pmol/mU)	14.1 (7.0, 17.8)	9.0 (7.9, 101.3)	>0.99

PA: primary aldosteronism; HTN: hypertension; K: potassium; BP: blood pressure; ARR: aldosterone to renin ratio; FU: follow-up.

Categorical data reported as number (percentage) and compared with Fischer’s exact test; quantitative data reported as median (interquartile range) and compared with the Mann-Whitney test (p significant if <0.05).

**Table 2:** Expression of enzymes responsible for retinoic acid biosynthesis in control adrenals (n = 11) and in APA (n = 48).

Gene name	Control adrenal	APA	*p*
Retinol Binding Protein 1	56.16 ± 7.30	81.77 ± 6.934	0.0673
Stimulated by Retinoic Acid 6	1.11 ± 0.13	1.485 ± 0.1561	0.3187
Retinol Dehydrogenase 5	1.05 ± 0.22	0.8240 ± 0.0730	0.2858
Retinol Dehydrogenase 11	19.30 ± 2.21	22.07 ± 1.466	0.2179
Retinol Dehydrogenase 12 (probe 2)	1.81 ± 0.35	5.704 ± 2.323	0.6776
Retinol Dehydrogenase 14	2.54 ± 0.32	3.914 ± 0.2692	0.0058
Aldehyde dehydrogenase 1 Family Member A1	24.28 ± 2.63	22.47 ± 2.589	0.3540
Aldehyde dehydrogenase 1 Family Member A3	2.72 ± 0.33	1.098 ± 0,1432	<0.0001
Cytochrome P450 Family 26 Subfamily B Member 1	1.69±0.37	0.8811 ± 0.1179	0.0189
Cellular Retinoic Acid Binding Protein 2	1.04 ± 0.31	1.076 ± 0.0825	0.2858

Data are presented as mean ± SEM and are compared with the Mann-Whitney test or t-test (p significant if <0.05). Data are expressed as log2.

### Significant disorganization of the adrenal cortex in 12 weeks old male and female Rarα^−/−^ mice

**Figure 2:**
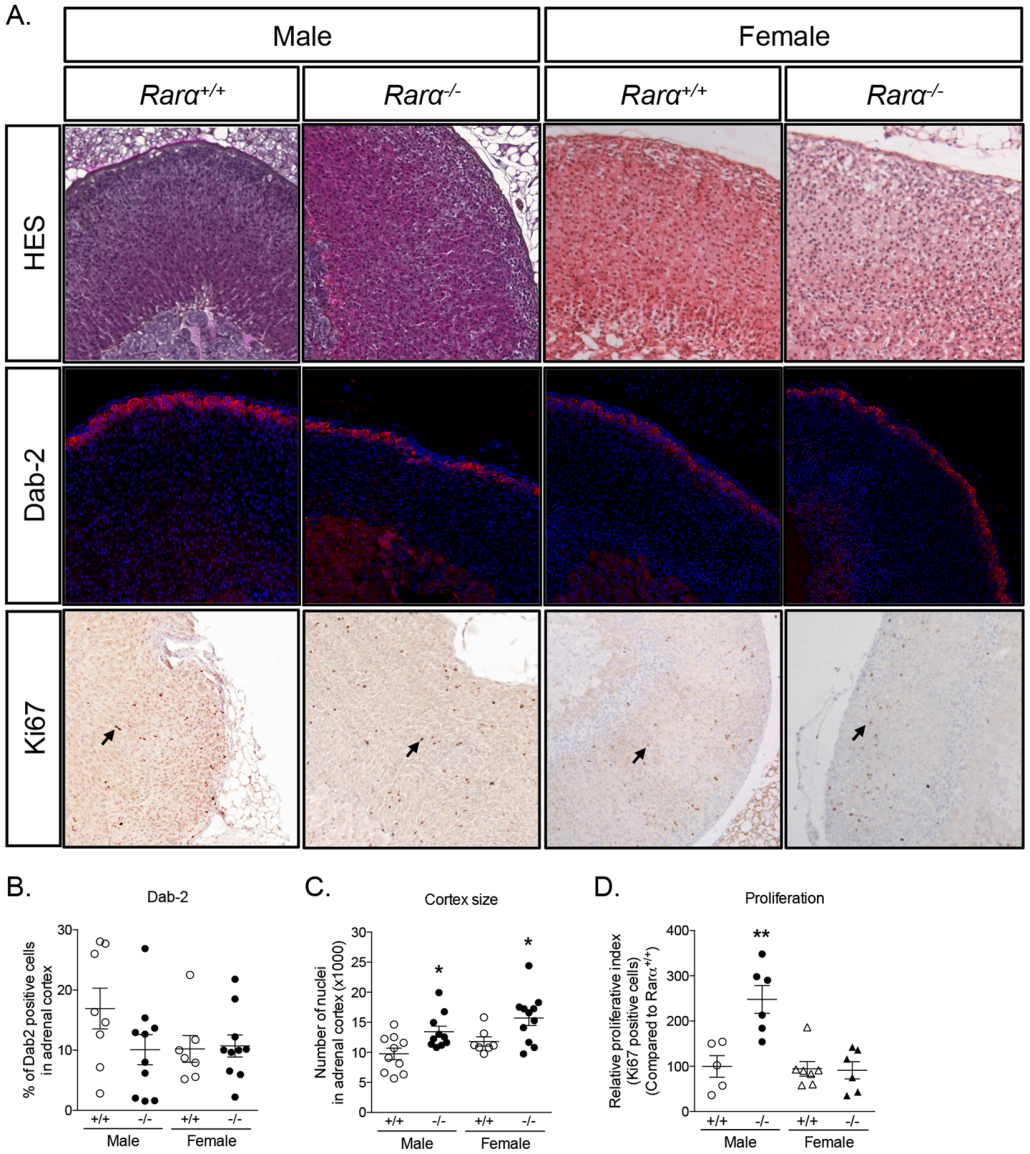
Rarα affects adrenal cortex morphology in 12 weeks old male and female mice. (**A**) Morphological characterization of adrenals from 12 weeks old *Rarα*^+/+^ and *Rarα*^−/−^ mice. HES staining, Dab-2 immunofluorescence and Ki67 immunohistochemistry were performed on adrenal sections from the indicated group of mice. (**B**) Number of Dab-2 positive cells in the cortex was determined in 7 to 11 animals of each genotype and sex using an automated molecular imaging platform (Vectra, Perkin Elmer) and is expressed as a percentage of total number of cells in the entire cortex area. (**C**) Number of nuclei in the adrenal cortex was determined in 7 to 11 animals of each genotype and sex using an automated molecular imaging platform (Vectra, Perkin Elmer). (**D**) Relative proliferative index of adrenals from male and female *Rarα*^+/+^ and *Rarα*^−/−^ mice. Ki67 positive cells were separately counted in the adrenal cortex in 5–6 animals per genotype and age. Values are presented as means ± SEM. *p<0.05; **p<0.01.

To evaluate the role of Rarα in adrenal cortex structure and function, we compared the adrenal phenotype of 12 weeks old male and female *Rarα*^+/+^ and *Rarα*^−/−^ mice. As previously described^32^, the *Rarα*^−/−^ mice exhibited growth retardation characterized by lower total body weight (Table 3). Relative adrenal weight of *Rarα*^+/+^ and *Rarα*^−/−^ was similar in male mice, while it was significantly increased in female *Rarα*^−/−^ mice (Table 3) compared to wild type littermates. HES staining revealed marked disorganization of the adrenal cortex, characterized by a loss of the radial organization of the zona fasciculata (ZF) in both male and female mice (Fig. 2A and Supplementary Fig. 2), with a conserved zona glomerulosa (ZG). Confirming the HES observation, the expression of Disabled-2 (Dab-2), a marker of ZG^33^, was not affected in *Rarα*^−/−^ mice (Fig. 2A,B), indicating that only the ZF was affected in both males and females. We also investigated the expression of 20-αHSD, a marker of the fetal X-zone^34^, which regresses at puberty in males and after the first gestation in females. No modification of 20-αHSD expression was observed in *Rarα*^−/−^ mice compared to wild type littermates, with the X-zone being visible in females but not in males in both genotypes (Supplementary Fig. 3), suggesting that the development and the regression of the fetal X-zone was unaffected. Total number of nuclei in adrenal cortex, indirectly reflecting adrenal cortex size, was significantly higher in 12 weeks old male and female *Rarα*^−/−^ mice (Fig. 2C). In males, this was associated with an increased proliferation, highlighted by an increase of Ki67 positive nuclei in the adrenal cortex in *Rarα*^−/−^ mice, (Fig. 2A,D). Although the proliferative index was not changed (Fig. 2A,D) in 12 weeks old female *Rarα*^−/−^ mice, the adrenal weight was significantly increased (Table 3) in addition to ZF expansion, suggesting that increased proliferation may have occurred at an earlier time point during development.

**Table 3:** Clinical characteristics of 12 and 52 weeks male and female *Rarα*^+/+^ and *Rarα*^−/−^ mice.

	Male					
	12 weeks			52 weeks		
	*Rarα* ^+/+^	*Rarα* ^−/−^	*p*	*Rarα* ^+/+^	*Rarα* ^−/−^	*p*
Total body weight (g)	28.12±0,59	26.45±0,70	**0**.**0135**	40.87±1.31	31.53±0.94	**<0**.**0001**
Left adrenal weight/total body weight (x1000)	1.531±0.112	1.678±0.144	0.7584	1.135±0.507	1.458±0.817	0.2736
Right adrenal weight/total body weight (x1000)	1.362±0.099	1.161±0.137	0.1112	1.173±0.684	1.460±0.687	0.1354
Left adrenal size (mm)	N.D.	N.D.		2.130±0.124	2.080±0.073	0.6663
Right adrenal size (mm)	N.D.	N.D.		2.015±0.068	2.069±0.066	0.7756
	**Female**					
Total body weight (g)	20.49±0.45	19.48±0.55	0.1612	29.97±1.75	23.25±0.71	**0**.**0005**
Left adrenal weight/total body weight (x1000)	2.044±0.091	2.474±0.188	**0**.**0332**	1.912±0.164	1.991±0.130	0.7108
Right adrenal weight/total body weight (x1000)	1.623±0.097	2.116±0.126	**0**.**0036**	1.506±0.105	1.784±0.162	0.1598
Left adrenal size (mm)	N.D.	N.D.		2.568±0.173	2.463±0.085	0.6095
Right adrenal size (mm)	N.D.	N.D.		2.430±0.156	2.260±0.132	0.3524

N.D., Not Determined. Quantitative data reported as mean ± SEM and compared with the Mann-Whitney test (p significant if <0.05).

**Figure 3:**
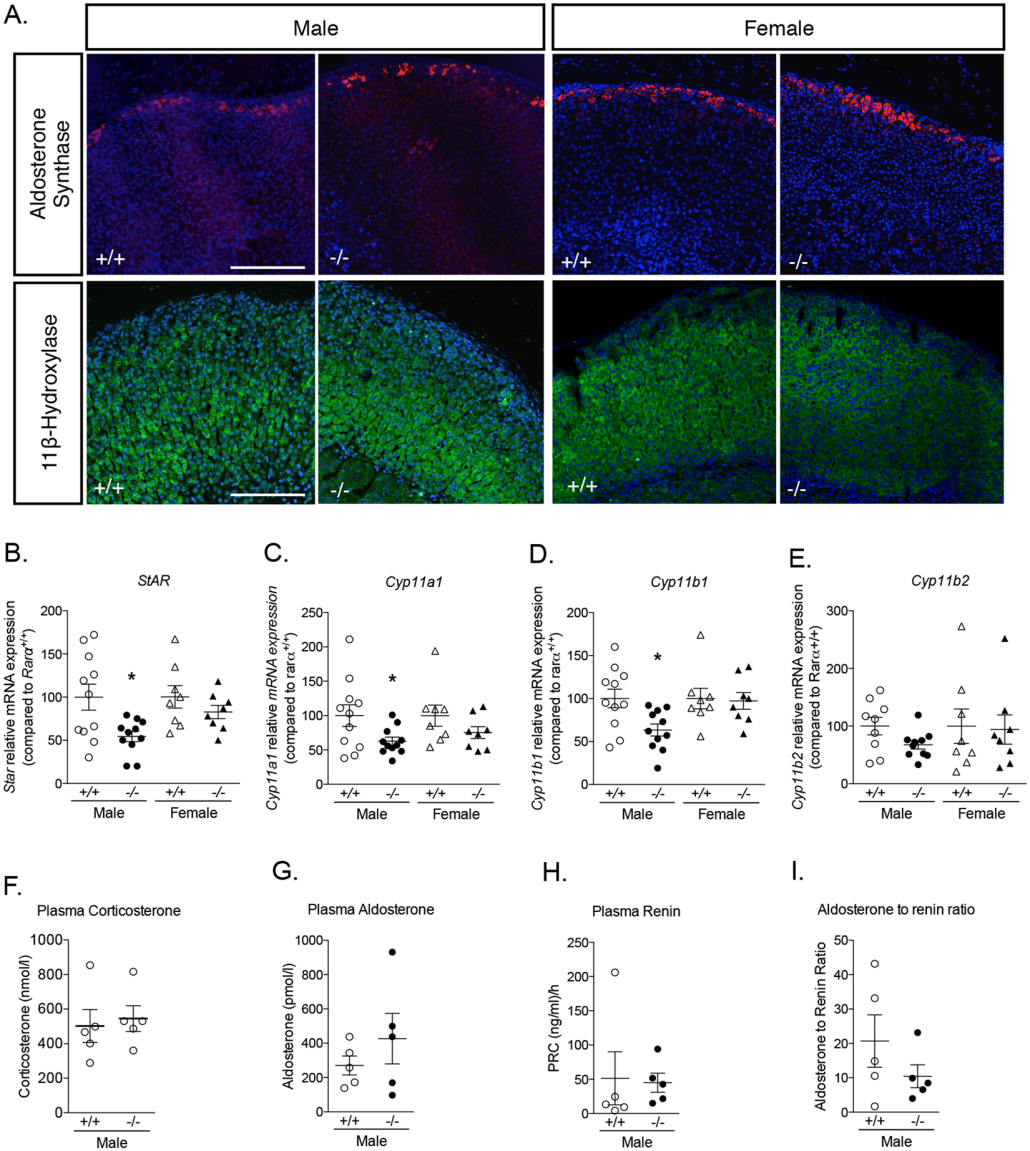
Impact of *Rarα* inactivation on adrenal steroidogenesis. (A) Expression of aldosterone synthase and 11β-hydroxylase in 12 weeks old male and female *Rarα*^+/+^ and *Rarα*^−/−^ mice. (**B**–**F**) Expression of steroidogenic genes in male and female *Rarα*^+/+^ and *Rarα*^−/−^ mice. mRNA expression of *Star* (**B**), *Cyp11a1* (**C**), *Cyp11b1* (**D**) and *Cyp11b2* (**E**) was assessed by RT-qPCR. RT-qPCR were performed on mRNA extracted from 6–11 adrenals from 12 weeks old male and female *Rarα*^+/+^ and *Rarα*^−/−^ mice. (**F**,**G**) Measure of plasma aldosterone (**F**) and corticosterone (**G**) concentration by mass spectrometry in male mice. (**H**,**I**) Plasma renin concentration (PRC) (**H**) and aldosterone to renin ratio (**I**). Measure of plasma aldosterone, plasma corticosterone and plasma renin were done on 5–6 animals per group. Values are presented as the mean ± SEM; p values were calculated using a Mann-Whitney test or *t*-test. For correlation, Pearson calculations were performed. *p < 0.05; **p < 0.01.

Aldosterone synthase expression was found to be restricted to the ZG in both *Rarα*^+/+^ and *Rarα*^−/−^ male and female mice (Fig. 3A). Interestingly, in both sexes, the localization of 11β-hydroxylase was not modified, despite ZF disorganization (Fig. 3A). However, the expression of genes coding for steroidogenic enzymes, including *Star* (Fig. 3B), *Cyp11a1* (Fig. 3C), and *Cyp11b1* (Fig. 3D) was reduced in 12 weeks male *Rarα*^−/−^ mice, while the expression of *Cyp11b2* was not significantly affected (Fig. 3E). Plasma aldosterone (Fig. 3F) and corticosterone levels (Fig. 3G), plasma renin concentration (Fig. 3H) and aldosterone to renin ratio (Fig. 3I) were not different in 12 weeks male *Rarα*^−/−^ mice.

### Modification of vessel architecture and extra cellular matrix composition in Rarα^−/−^ mice

**Figure 4:**
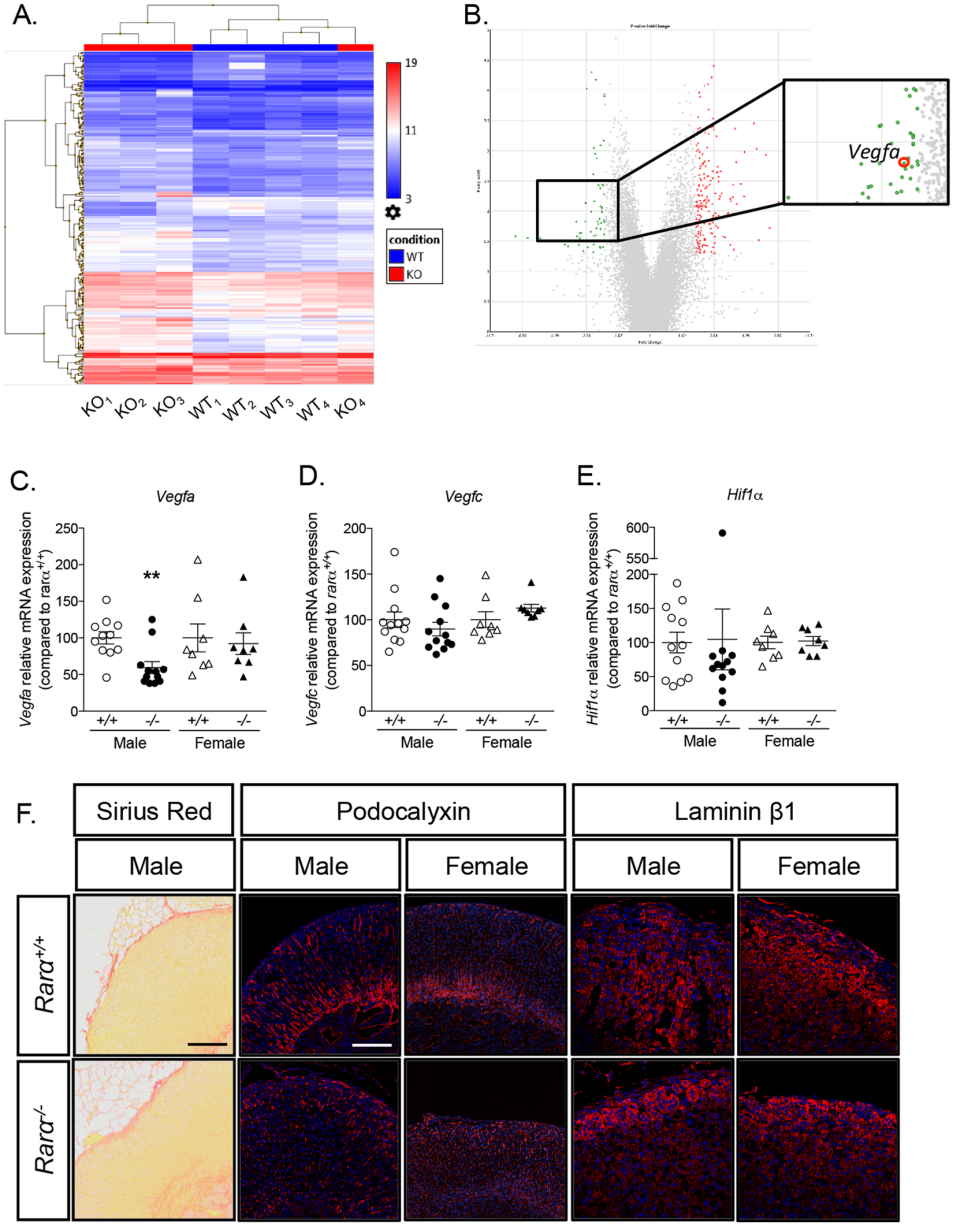
*Rarα* affects vessel architecture and extra cellular matrix composition. (**A**) Hierarchical clustering of samples using the 243 differentially expressed genes in adrenals from 12 weeks old *Rarα*^+/+^ and *Rarα*^−/−^ male animals (4 animals per group). (**B**) Volcano plot showing the differentially expressed genes in 12 weeks old *Rarα*^+/+^ and *Rarα*^−/−^ animals. The x-axis is the fold change between the two conditions; the adjusted p value based on –log_10_ is reported on the y-axis. Genes significantly different are highlighted as green (down-regulated in *Rarα*^−/−^ mice) or red (up-regulated in *Rarα*^−/−^ mice) dots. (C, D, E) The expression of genes involved in angiogenesis, *Vegfa* (**C**) and *Vegfc* (**D**) and in hypoxia, *Hif1α* (**E**) was investigated by RT-qPCR on mRNA extracted from 6–11 adrenals from 12 weeks old male and female *Rarα*^+/+^ and *Rarα*^−/−^ mice. (**F**) The vascular architecture was assessed by Sirius red staining and podocalyxin immunofluorescence and extra cellular matrix integrity by laminin β1 immunofluorescence. Values are presented as the mean ± SEM; p values were calculated using a Mann-Whitney test. **p < 0.01.

To gain mechanistic insight into the activity of *Rarα* in the adrenal cortex, microarray analysis was performed on adrenals from four *Rarα*^+/+^ and four *Rarα*^−/−^ male mice. Hierarchical clustering allowed separating three out of four *Rarα*^−/−^ mice from WT mice (Fig. 4A,B). Among the 243 statistically significantly differentially expressed genes, 184 genes (75.72%) were found to be upregulated and 59 (24.28%) down-regulated. Gene Ontology analyses allowed the identification of specific molecular functions and biological processes enriched in 12 weeks *Rarα*^+/+^ versus *Rarα*^−/−^ mice (Supplementary Fig. 2), which are mainly involved in specific metabolic processes, such as lipid, carboxylic acid or fatty acid metabolism. Interestingly, among the differentially regulated genes, *Vegfa* mRNA expression was found to be downregulated in *Rarα*^−/−^ mice compared to *Rarα*^+/+^ mice (Fig. 4B, Supplementary Table 12). The down-regulation of *Vegfa* mRNA expression was confirmed by RT-qPCR in male mice only (Fig. 4C). The expressions of *Vegfc*, involved in angiogenesis and lymphangiogenesis, and of *Hif1α*, a marker of hypoxia, were unchanged (Fig. 4D,E). Sirius red staining, performed in male mice, revealed dilatation of vessels (Fig. 4F). In addition, podocalyxin staining of endothelial cells revealed disorganization of vessel architecture in both male and female *Rarα*^−/−^ mice (Fig. 4F). Expression of fibronectin 1, microfibrillar associated protein 2 and 5 (Mfap2 and Mfap5) and of collagen 3α1, all components of the extracellular matrix (ECM), was found to be up-regulated in male *Rarα*^−/−^ mice (Supplementary Table 12). To evaluate if *Rarα* inactivation alters ECM structure, laminin β1 staining was performed. Laminin β1, a component of the basement membrane of the ECM, was expressed homogeneously throughout the adrenal cortex in both male and female *Rarα*^+/+^ mice (Fig. 4F). In *Rarα*^−/−^ mice, expression of laminin β1 showed a gradient, with higher expression in the ZG than in the inner part of the ZF in male but not in female mice (Fig. 4F). Since expression of laminin β1 was not modified in microarray analyses, this suggests that modification of the expression of other components of the ECM may alter proper laminin β1 localization.

### Inhibition of Wnt signaling in young Rarα^−/−^ male mice

**Figure 5:**
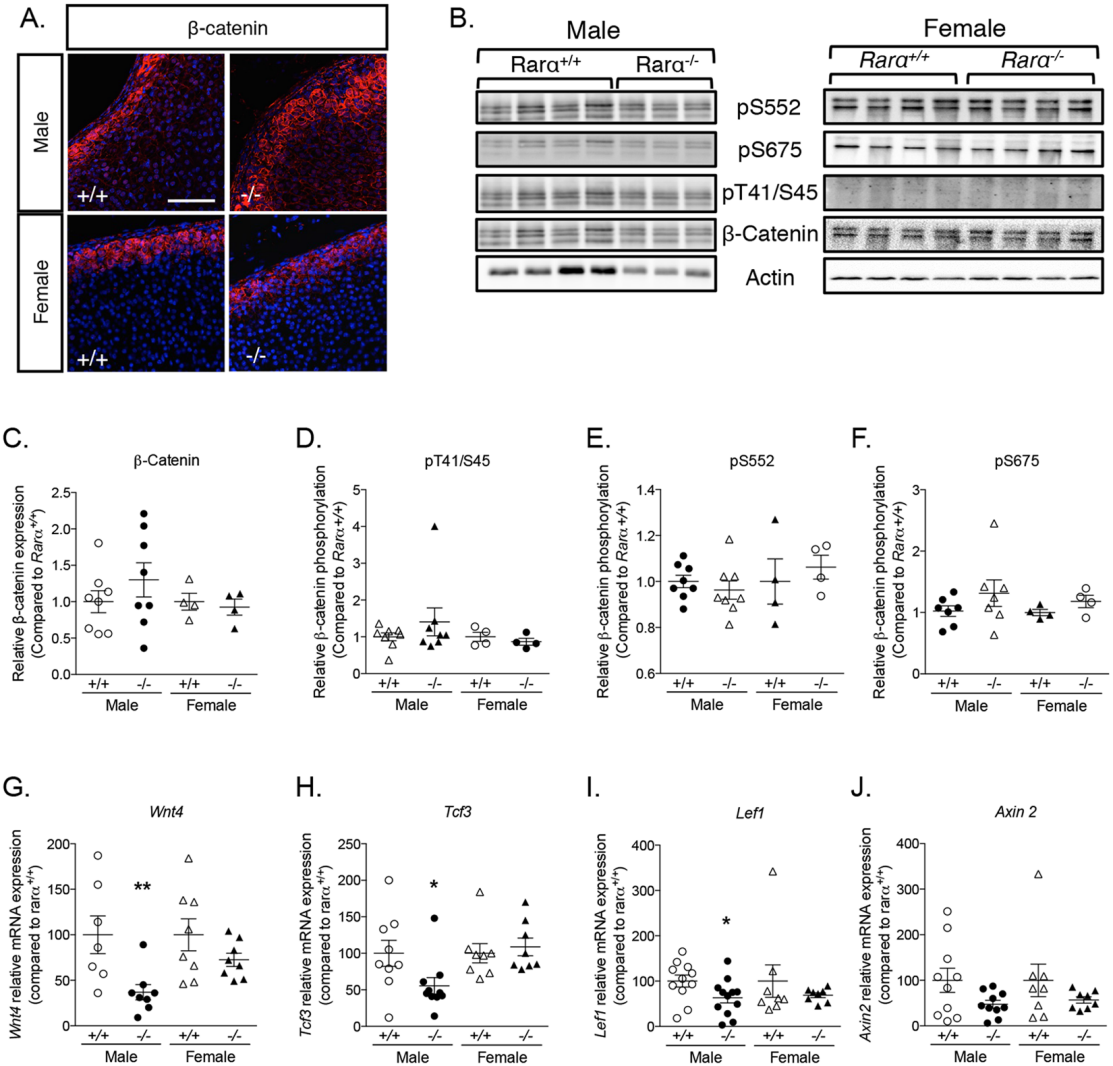
*Rarα* inactivation alters Wnt signaling pathway in 12 weeks old male mice. (**A**) Expression of β-catenin was evaluated by immunofluorescence in 12 weeks old male and female *Rarα*^+/+^ and *Rarα*^−/−^ mice. (**B**) Expression and phosphorylation of β-catenin in response to *Rarα* invalidation. Proteins were extracted from total adrenal and submitted to western blot analysis. Phosphorylation/dephosphorylation in activating (pS552 and pS675) and inactivating (pT41/S45) residues and total expression of β-catenin was investigated. (**C**–**F**) Quantification of β-catenin expression (**C**) and of phospho-specific signals in inactivating (**D**) and activating (**E**,**F**) residues was performed in *Rarα*^+/+^ and *Rarα*^−/−^ adrenal. (G-J) The expression of *Wnt4* (**G**), *Tcf3* (H), *Lef1* (**I**) and *Axin2* (**J**) was investigated by RT-qPCR on mRNA extracted from 6 to 11 adrenals from *Rarα*^+/+^ and *Rarα*^−/−^ male and female mice. Values are presented as mean ± SEM; p values were calculated using a Mann-Whitney test or *t*-test. *p < 0.05; **p < 0.01.

Wnt/β-catenin and PKA signaling are central regulators of adrenal cortex development and function, with a fine equilibrium between the activation of Wnt/β-catenin pathway in the ZG and of PKA signaling in the ZF. To gain mechanistic insight into the changes responsible for structural modifications of the adrenal cortex in *Rarα*^−/−^ mice, we thus explored Wnt/β-catenin signaling (Fig. 5). Whereas in female *Rarα*^+/+^ animals, β-catenin expression was restricted to the ZG with membranous, cytoplasmic and nuclear localization, in male mice low expression was also detected in the external part of the ZF (Fig. 5A). In male *Rarα*^−/−^ mice, high expression of β-catenin was observed in the outer layer of the adrenal cortex, extending into the ZF (Fig. 5A); the localization of β-catenin was unaffected in female *Rarα*^−/−^ mice (Fig. 5A). Western blot performed on whole adrenals showed that the expression of β-catenin was not significantly increased in male and female *Rarα*^−/−^ mice compared to wild type littermates (Fig. 5B,C), although a large variation of its expression was observed in male *Rarα*^−/−^ mice (Fig. 5C) consistent with the extended localization observed in some animals. β-catenin phosphorylation is a marker of its activation state; phosphorylation on inactivating (T41/S45) (Fig. 5D) or activating (S552, S675) (Fig. 5E,F) residues was not affected by Rarα expression, indicating absence of activation of the canonical Wnt/β-catenin pathway in *Rarα*^−/−^ mice. This was also supported by unchanged mRNA expression of *Axin2* (Fig. 5J), a target gene of the canonical Wnt/β-catenin pathway. In contrast, mRNA expression of *Wnt4* (Fig. 5G), *Tcf3* (Fig. 5H) and *Lef1* (Fig. 5I), effectors and targets of non-canonical Wnt signaling, was reduced in adrenals from 12 weeks old *Rarα*^−/−^ male mice. Modification of the expression of Wnt4 (Fig. 5G), a ligand involved in non-canonical Wnt signaling, of Frizzled-2, a transmembrane receptor of the non-canonical pathway (Supplementary Table 12) and of Tcf3 (Fig. 5H), a transcription factor that can act as a transcriptional repressor independently of β-catenin binding, suggests that the non-canonical Wnt signaling pathway is altered in *Rarα*^−/−^ mice.

Since *Rarα*^−/−^ mice show significant abnormalities of the ZF, we also explored protein kinase A (PKA) activation, which plays an important role in adrenal cortex development and function. It has been proposed that PKA prevents ZG differentiation through Wnt pathway inhibition, suggesting that PKA activation in the ZF is a key driver of Wnt inhibition^35^. PKA is the main kinase responsible for the phosphorylation of specific transcription factors including the cAMP response element-binding protein (CREB)^36^. p-CREB was detected in the entire cortex in *Rarα*^+/+^ and *Rarα*^−/−^ (Supplementary Fig. 4), where its expression was detected in >70% of the cells (Supplementary Fig. 4). Western blot analysis of CREB and p-CREB expression, performed on whole adrenals, revealed no change in total CREB expression or in its phosphorylation status depending on genotype (Supplementary Fig. 4). mRNA expression of *Prkar1a* and *Prkaca*, the genes coding for the regulatory and catalytic subunit of PKA, was reduced in *Rarα*^−/−^ animals (Supplementary Fig. 4); given the concomitant modification of both subunits, this did not affect the expression of *S100a4*, a target gene of PKA activation in the adrenal^37^ (Supplementary Fig. 4).

### Significant disorganization of the adrenal cortex persists with aging

**Figure 6:**
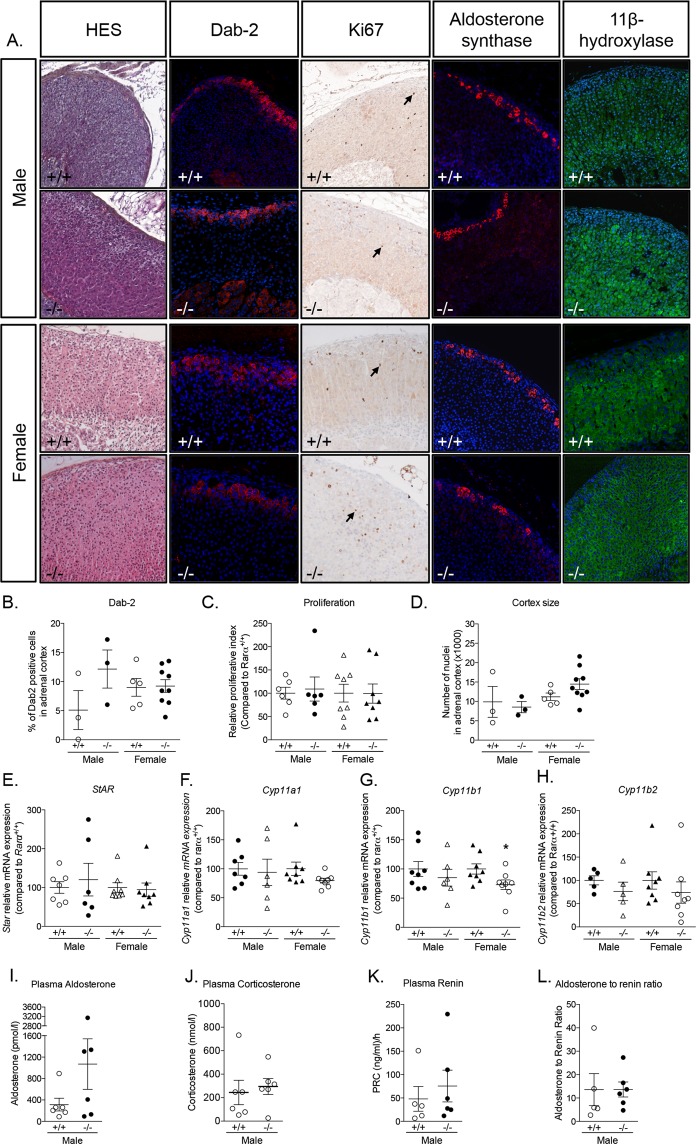
Adrenal cortex disorganization persists with aging in *Rarα*^−/−^ mice. (**A**) Morphological characterization of adrenals from 52 weeks old male and female *Rarα*^+/+^ and *Rarα*^−/−^ mice. HES staining, Dab-2, aldosterone synthase and 11β-hydroxylase immunofluorescence and Ki67 immunohistochemistry were performed. (**B**) Number of Dab-2 positive cells in the cortex was determined in 3 to 9 animals of each genotype and sex using an automated molecular imaging platform (Vectra, Perkin Elmer) and is expressed as a percentage of total number of cells in the entire cortex area. (**C**) Relative proliferative index of adrenals from male and female *Rarα*^+/+^ and *Rarα*^−/−^ mice. Ki67 positive cells were separately counted in the adrenal cortex in 5–6 animals per genotype. (**D**) Number of nuclei in the adrenal cortex was determined in 3 to 9 animals of each genotype and sex using an automated molecular imaging platform (Vectra, Perkin Elmer). (**E**–**H**) Expression of steroidogenic genes in male and female *Rarα*^+/+^ and *Rarα*^−/−^ mice. mRNA expression of *Star* (**E**), *Cyp11a1* (**F**), *Cyp11b1* (**G**) and *Cyp11b2* (**H**) was assessed by RT-qPCR. RT-qPCR were performed on mRNA extracted from 6–8 adrenals from 52 weeks old male and female *Rarα*^+/+^ and *Rarα*^−/−^ mice. (**I**,**J**) Measure of plasma aldosterone (I) and corticosterone (**J**) concentration by mass spectrometry in male mice. (**K**,**L**) Plasma renin concentration (PRC) and aldosterone to renin ratio. Measure of plasma aldosterone, plasma corticosterone and plasma renin were done on 5–6 animals per group. Values are presented as means ± SEM. *p < 0.05; **p < 0.01.

As *Rarα* was identified as associated with abnormal cell proliferation and nodule formation in APA, we evaluated the evolution of adrenal abnormalities with aging by investigating the adrenal phenotype of *Rarα*^−/−^ animals at 52 weeks of age (Fig. 6 and Supplementary Fig. 5). Growth retardation was more pronounced in 52 weeks old compared to 12 weeks old mice, with smaller weight for both male and female *Rarα*^−/−^ compared to *Rarα*^+/+^ mice; the relative adrenal weight was similar in both genotypes (Table 3). Adrenal morphology and size, evaluated by magnetic resonance imaging, was also similar in both genotypes and sexes (Table 3). Similar to 12 weeks old *Rarα*^−/−^, we observed loss of radial organization of the ZF with a conserved ZG in both male and female mice at 52 weeks of age (Fig. 6A,B), and absence of the fetal X-zone in both genotypes (Supplementary Fig. 3). However, at 52 weeks, the proliferation index and adrenal cortex size were similar in *Rarα*^−/−^ mice compared to their wild type littermates (Fig. 6C,D). No differences were observed in the localization of aldosterone synthase or 11 β-hydroxylase expression (Fig. 6A). Unlike 12 weeks old animals, there was no difference in the expression of steroidogenic genes such as *Star* (Fig. 6E), *Cyp11a1* (Fig. 6F), and *Cyp11b2* (Fig. 6H) in *Rarα*^−/−^ mice; a slightly decreased expression of *Cyp11b1* (Fig. 6G) was observed in female mice only. Again, plasma aldosterone (Fig. 6I) and corticosterone (Fig. 6J) levels, plasma renin concentration (Fig. 6K) and aldosterone to renin ratio (Fig. 6L) were not different in *Rarα*^−/−^ mice. However, 50% of male *Rarα*^−/−^ mice showed high plasma aldosterone concentrations (up to 1000 pmol/l), which were associated with higher plasma renin concentration (Fig. 6K).

Vessel dilatation (Sirius red) and disorganization (podocalyxin) as well as abnormal laminin β1 localization were still present in both male and female mice (Supplementary Fig. 5), but expression of *Vegfa* was not modified in *Rarα*^−/−^ mice at 52 weeks of age (Supplementary Fig. 5). β-catenin was localized to the ZG (Supplementary Fig. 5), with an extended expression in ZF that could be observed in restricted areas in some animals (data not shown). Consistently with this observation, despite similar β-Catenin protein levels in *Rarα*^+/+^ and *Rarα*^−/−^ mice (Supplementary Fig. 5), a large variation in its expression was observed (Supplementary Fig. 5). Again, β-Catenin phosphorylation on activating (pS552 and pS675, Supplementary Fig. 5) or inactivating (pT41/S45, Supplementary Fig. 5) residues were unchanged, indicating again that canonical Wnt/β-Catenin signaling was not modified in 52 weeks old *Rarα*^−/−^ mice. In contrast to 12 weeks old *Rarα*^−/−^ mice, only the expression of *Lef1* was reduced in 52 weeks old male *Rarα*^−/−^ mice (Supplementary Fig. 5), while the expression of *Wnt4* (Supplementary Fig. 5), *Tcf3* (Supplementary Fig. 5) and *Axin 2* (Supplementary Fig. 5) was similar in both genotypes. Similar to 12 weeks old mice, PKA signaling was unaltered at 52 weeks of age (Supplementary Fig. 4). However, p-CREB expression was reduced (<20% of cells, Supplementary Fig. 4) and found to be restricted to the external part of the adrenal cortex, mainly the ZG, in both *Rarα*^+/+^ and *Rarα*^−/−^ animals at that age (Supplementary Fig. 4).

Microarray analysis performed on adrenals from four *Rarα*^+/+^ and four *Rarα*^−/−^ 52 weeks old male mice revealed that 63 genes (60%) were upregulated and 42 (40%) down-regulated in *Rarα*^−/−^ mice. Despite the low number of differentially regulated genes, hierarchical clustering allowed separating adrenals form *Rarα*^+/+^ and *Rarα*^−/−^ mice (Supplementary Fig. 6). The expression of some genes involved in retinoic acid biosynthesis was found to be increased (alcohol dehydrogenease 1 and 7), possibly to compensate for the lack of Rarα expression (Supplementary Table 13). Furthermore, the expression of *Mrap* (Melanocortin 2 receptor accessory protein) was also found to be upregulated in these mice. *Mrap* has recently been shown to play a role in adrenal cortex zonation through the modulation of adrenal progenitor cell differentiation. Absence of *Mrap* was found to be associated to Wnt4/β-Catenin pathway dysregulation in ZG cells, showing an involvement of both the canonical and the non-canonical Wnt/β-Catenin pathway in adrenal gland zonation^38^.

## Discussion

Several germline and somatic mutations cause excessive aldosterone production in PA. Although the functional link between those mutations and aldosterone production has been clearly established, the mechanisms leading to nodule formation appear to be complex and to partly involve different mechanisms^39^. Here we report the identification of RARα as being a key element in the regulation of adrenal cortex structure and cell proliferation. Inactivation of *Rarα* in mice led to significant structural disorganization of the adrenal cortex in male and female, with increased adrenal cortex size in female mice and increased cell proliferation in males, but no major modifications of aldosterone production. This disorganization is associated with modifications of the vessel architecture and ECM due to decreased Vegfa expression and modifications in ECM components. On the molecular level, *Rarα* inactivation leads to inhibition of the non-canonical Wnt signaling, without affecting the canonical Wnt Pathway nor PKA signaling. These molecular abnormalities were detected at 12 weeks in male mice only, suggesting that in female mice they may have occurred earlier in adrenal cortex development. At 52 weeks of age, the marked disorganization of the adrenal cortex persists in absence of molecular abnormalities in Wnt or Vegf signaling.

RAR belongs to the super-family of nuclear receptors; there are three subtypes of RAR: α (*NR1B1*), β (*NR1B2*) and γ (*NR1B3*). RAR functions as a ligand dependent transcription factor, which heterodimerizes with another receptor of the same family, the retinoic X receptor (RXR). RXR/RAR heterodimers bind to specific DNA sequences, the retinoic acid (RA) response element (RAREs) composed of two direct repeats of a core hexameric motif (A/G) G [G/T] TCA located in the regulatory regions of target genes^40,41^. In the absence of ligand, RARs are bound to RAREs in association with large protein complexes, which maintain chromatin in a condensed and repressed state^42^. Ligand binding is facilitated by association of RAR with cellular RA binding proteins. This binding induces conformational changes in the ligand-binding domain of the receptor, resulting in corepressor release and coordinated recruitment of a series of coregulator complexes.

RA mediated transcription plays critical roles in a variety of biological processes, including development, reproduction, immunity, organogenesis and homeostasis^43^. Moreover RARs are involved in cancer development, due to mutations, fusions to other proteins, altered expression or aberrant post-translational modifications. These alterations result in modified function and disruption of homeostasis, due to the capacity of RAR to regulate growth and differentiation. In human myeloid leukemia, the RARα gene is the target of chromosomal rearrangements resulting in the production of fusion proteins that allow the cells to continue to proliferate and/or prevent the terminal differentiation seen in normal myelocytes^44^. Considering the associations between RAR and tumorigenesis, RAR are considered as tumor suppressor. In some cases, RAR have been correlated with survival instead of growth arrest. Recently, proteomic characterization of adrenal gland embryonic development suggested a role of the retinoic acid receptor pathway in the early stages of adrenal gland development^45^. Moreover, transcriptome analysis identified LPS/IL-1 mediated inhibition of RXR function pathway in the top 5 canonical pathways associated with genes differentially regulated in APA compared with adjacent ZG, suggesting a role of RXR pathways in APA^46^.

RA is a derivative of vitamin A (retinol), which is provided exclusively by diet. Vitamin A is converted into retinal by the action of alcohol dehydrogenases and short chain dehydrogenases/reductases. Then retinal is oxidized into RA by retinaldehyde dehydrogenase^47^. In mice, the half-life of RA has been estimated to be about 0.5 hours whereas retinol′s half-life is longer (3.6 hours)^48^, suggesting that RA could be produced locally in target tissues instead of being transported. All components of retinoic acid biosynthesis were identified in human and mouse adrenals in this study, supporting RA biosynthesis and action in the adrenal gland. Our data support a role for Rarα signaling in regulating cell proliferation: indeed, 12 weeks old male *Rarα*^−/−^ mice show an increased proliferative index compared to wild type littermates. Despite no changes in proliferative index observed in female mice at 12 weeks of age, their adrenal weight was increased, suggesting that a proliferative phase may have occurred at earlier stages of adrenal development^24^.

The Wnt/β-catenin pathway is known to play an important role in embryonic development, stem cell maintenance, and differentiation in many tissues^22,49–52^. Depending on the Wnt-receptor complex formed, a β-catenin dependent (canonical pathway) or independent response (non-canonical pathway) is activated. In the canonical pathway, the binding of Wnt ligands (Wnt1, Wnt2, Wnt3a, Wnt8a, Wnt8b…) to specific frizzled – Low-density lipoprotein receptor-related protein (Lrp) complex leads to the dissociation of β-catenin from the degradation complex and its translocation into the nucleus where it induces the expression of specific target genes (*Lef1*, *Axin 2*, *c-jun*, *Cyclin D1*, *c-myc*…)^22^ through binding to specific transcription factors (T-cell factor/lymphoid enhancer factor, Tcf/Lef). The Tcf/Lef family is composed of four members Tcf1, Lef1, Tcf3 and Tcf4. Whereas Tcf1 and Lef1 mediate transcriptional activation of Wnt target genes, Tcf3 acts as a transcriptional repressor, independently of β-catenin binding^53^. In addition to this canonical Wnt/β-catenin pathway, a non-canonical Wnt pathway has been described involving binding of other Wnt ligands (Wnt4, Wnt5a, Wnt5b, Wnt6, Wnt7a, Wnt7b and Wnt11) to frizzled receptors (Fzd2, Fzd3, Fzd4 and Fzd6), which signals through the c-Jun N-terminal kinase (JNK) and the Ca^2+^ signaling pathways^22,54^. Activation of the non-canonical pathway replaces Tcf1 by Tcf3 in the Tcf/Lef complex, changing the transcriptional program induced by Tcf/Lef binding to DNA^55^.

In the adrenal cortex, β-catenin expression is restricted to the ZG, suggesting a role for this pathway in the development of the adrenal cortex^56^. In *Wnt4*-deficient mice, a decrease in ZG cell number has been reported, resulting in a decrease of *Cyp11b2* mRNA expression and aldosterone production. These mice also show ectopic expression of adrenal-like cells in the gonads, probably due to abnormal adrenocortical progenitor cell migration during development, suggesting a role for Wnt4 in cell sorting during development^57^. In our model, the expression of *Wnt4* was highly correlated to the expression of *Cyp11b2* in both male (r = 0.6015, p = 0.0064) and female (r = 0.5663, p = 0.0007) mice, supporting the role of Wnt4 in the regulation of *Cyp11b2* expression. Similarly, the targeted disruption of β-catenin in steroidogenic Sf-1-expressing cells causes adrenal aplasia in newborn mice, further supporting a role for the Wnt/β-catenin pathway early in adrenal development^56^.

The marked disorganization of adrenal cortex structure observed in *Rarα* knockout mice, was associated to reduced expression of *Lef1*, *Tcf3*, *Wnt4* and increased expression of *Fzd2* (Supplementary Table 12), all involved in non-canonical Wnt signaling, in 12 weeks old *Rarα*^−/−^ male mice. In contrast, β-catenin phosphorylation, which reflects the activation status of the canonical Wnt/β-catenin pathway, was unchanged. Interactions between the Wnt pathway and retinoic acid receptor signaling have been reported in some studies. Hence, in mouse embryonic stem cells, retinoic acid can concomitantly activate the non-canonical and inhibit the canonical Wnt pathway^55^. Similarly, the expression of *Lef1*, known to be regulated by the canonical Wnt pathway, can also be modulated by the non-canonical pathway^58^. In chondrocytes, retinoic acid, through RARα/RXRα activation, plays an important role in terminal differentiation through BMP2 and Wnt4 modulation^59^. More interestingly, retinoic acid has been proposed to inhibit β-catenin/Tcf activity downstream of β-catenin by inducing expression of secreted factors such as Wnt4 or Wnt11^60^. Increased expression of Tcf3, concomitantly with decreased expression of Tcf1 enhances the ability of Tcf3 to bind to specific Wnt response element, inducing changes in the transcriptional program of the cells^55^. Therefore, our results suggest that Rarα could contribute to the structural organization of the adrenal cortex through modification of the expression of Wnt4 and Tcf3, thus modulating the non-canonical Wnt pathway. Recently, downregulation of retinoic acid signaling (liver and retinoid X receptors LXR/RXR) was reported in adrenals of *Siah1* KO mice. *Siah1* KO mice exhibit severe disorganization of adrenal glands as well as increased aldosterone and corticosterone levels and vessel dilatation. *Siah1* codes for an E3 ubiquitin-protein ligase mediating ubiquitination and subsequent proteasomal degradation of target proteins, including PIAS1. On a molecular level, the stabilisation of PIAS1 leads to the enhancing sumoylation of Sf1^61^ and LXR^62^. Whereas, Sf1 sumoylation results in increased steroidogenesis, modification of retinoic acid signaling could contribute to adrenal cortex disorganization as well as vessel dilatation.

PKA signaling has been proposed to play a role in cell conversion from ZG to ZF and in maintenance of adrenal cortex zonation, acting through the modulation of the Wnt/β-catenin pathway, by inhibiting Wnt4 expression^35^. In 12 weeks old male *Rarα*^−/−^ mice, the mRNA expression of both the regulatory and catalytic subunits of PKA (*Prkar1a* and *Prkaca* respectively) was downregulated; however CREB phosphorylation was not affected, neither was the expression of *S100a4*, a specific adrenal target gene of PKA^37^, suggesting that PKA activity was not affected. Therefore, the observed inhibition of the Wnt pathway does not appear to be due to modifications of PKA activity, but rather to a direct effect of Rarα.

Transcriptome analysis of adrenals from 12 and 52 weeks old *Rarα*^+/+^ and *Rarα*^−/−^ mice, combined with specific staining revealed a decrease in *Vegfa* expression as well as abnormal vascular architecture. This was associated with a differential pattern of expression of laminin β1, which was present in the entire adrenal cortex in *Rarα*^+/+^ mice, whereas a gradient of expression was observed in *Rarα*^−/−^ mice with higher expression in the ZG than in the inner part of the ZF. The extracellular environment, in particular the vasculature and ECM, plays an important role in adrenal cortex structure and function, by modulating proliferation, migration, differentiation and survival of surrounding cells, but also steroidogenesis^63,64^. The ECM consists of the basement membrane and the interstitial ECM. The ECM is composed of polysaccharides and extracellular proteins such as collagens, laminins and fibronectin, but also some bioactive compounds (growth factors, enzymes, chemoattractants and morphogens), that form a three-dimensional network^65^. Laminins were found to be uniformly distributed in human adrenal cortex^64,66^; they have been proposed to play a role in adrenocortical cell migration through their chemotactic and haptotactic properties^66^. Interestingly, presence of RARα response elements has been identified in the laminin β1 promoter indicating regulation of its expression by retinoic acid^67^. Different studies reported that retinoic signaling participates in the regulation of the expression of ECM proteins as well as of cell membrane ECM receptors in physiological and pathophysiological conditions^68^. The modification of expression of some components of the ECM such as Fibronectin 1, MFAP2, MFAP5 and of Collagen 3α1, associated to the modification of the pattern of expression of laminin β1 observed in the adrenal cortex of *Rarα*^−/−^ mice strongly suggest a rearrangement of the ECM in absence of Rarα expression. These alterations of ECM composition could contribute to the disorganization of the adrenal cortex observed in our model, by modulating adrenocortical and vascular cell migration.

The adrenal gland is a highly vascularized tissue, each cell being in contact with at least one endothelial cell, allowing rapid release of hormones into the blood stream. Blood vessels present a centripetal pattern from superficial arteries to the central vein^69^, following the radial organization of the adrenal cortex^70^. A characteristic feature of adrenal glands is the high expression of VEGF, which is produced by steroidogenic cells and is regulated by ACTH^71^. Interestingly, the Wnt pathway has also been involved in angiogenesis and vessel remodeling. It has been proposed that Wnt inhibition leads to vessel stability or regression whereas its activation could lead to angiogenesis or vessel remodeling^72,73^. VEGF plays a central role to optimize vessel growth^74^; more importantly, many studies reported an effect of retinoic acid on VEGF expression; however depending on the cell models and the experimental conditions retinoic acid inhibits or stimulates VEGF expression^75,76^. Hence, the adrenal phenotype observed in *Rarα*^−/−^ mice could be explained by a direct effect of deficient Rarα signaling on Vegfa signaling and Laminin β1 expression or by the inhibition of Wnt signaling, that could in turn also affect Vegf signaling. These modifications of the extracellular microenvironment in *Rarα* KO mice suggest that Rarα may affect the interplay between steroidogenic cell differentiation and migration, vasculature and extracellular matrix.

The development of specific mouse models in recent years has allowed a better understanding of the mechanism of male adrenal cortex development^65,77^; however little is still known in female mice. Sex differences in adrenal physiology have been demonstrated by Heitzmann and co-workers^27^. Invalidation of the Task1 potassium channel leads to abnormalities of adrenal zonation and PA development, which occurs only in female mice after puberty. Testosterone treatment before puberty in female Task1^−/−^ mice leads to normal adrenal zonation. Similarly, castration of male Task1^−/−^ mice leads to abnormal zonation of the adrenal cortex similar to female Task1^−/−^ mice. The comparison of gene expression profiles of male and female adrenals allowed the identification of a set of genes that are modulated according to sex and hormonal treatments^26^. These studies highlighted the critical role for sexual hormones in adrenal gland zonation and the existence of a sexual dimorphism. Comparison of the adrenal phenotype of male and female Rarα^−/−^ mice revealed sex-specific molecular differences in young mice despite similar morphological abnormalities in adrenal gland development. This sexual dimorphism could be due to a different timing of the action of Rarα in adrenal development, with Rarα signaling occurring earlier in female than in male. As suggested by the adrenal phenotype of Task1^−/−^ mice, sexual hormones, such as estrogen and androgens, may play a role in adrenal development and modulate the effect of Rarα inactivation.

**Figure 7:**
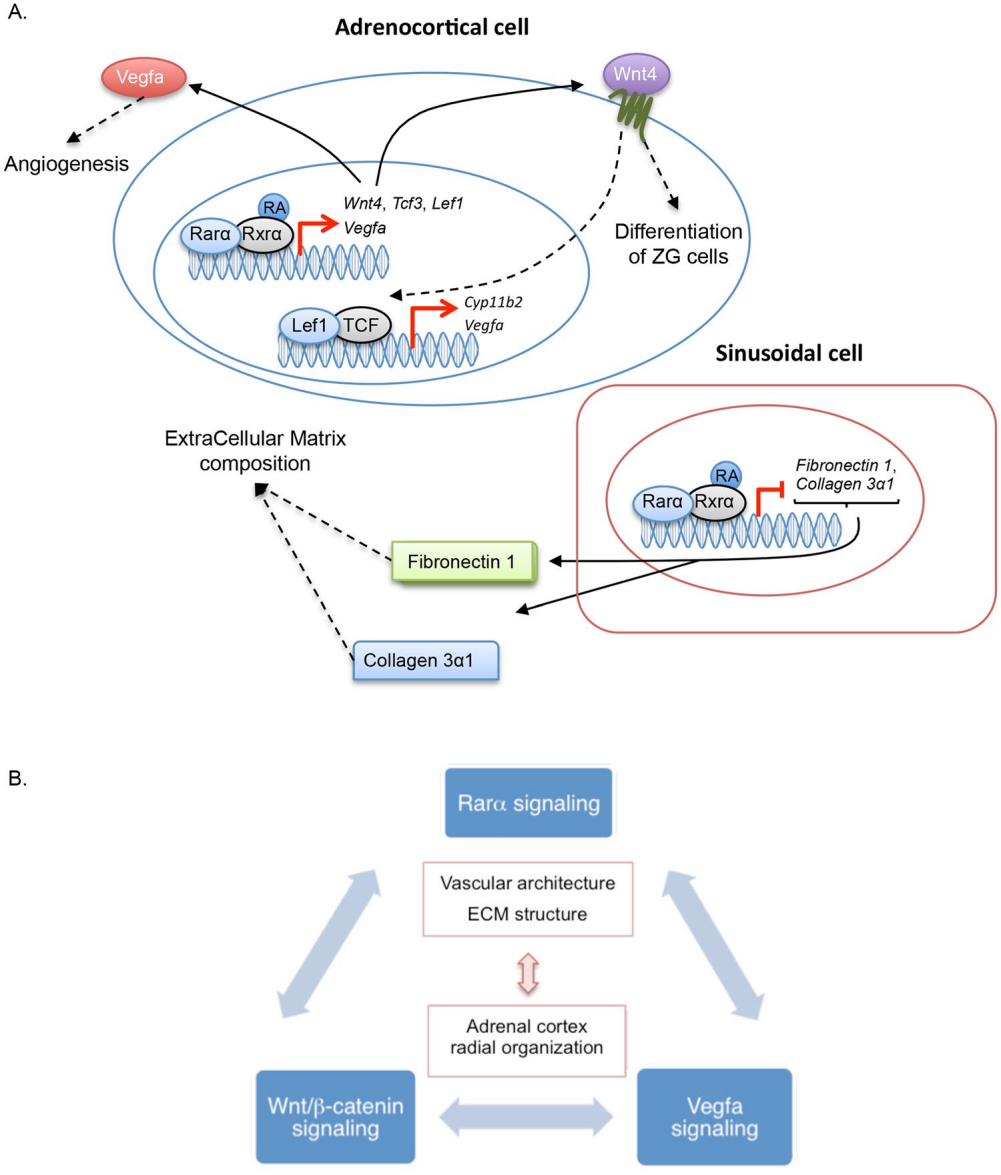
Proposed model for the role of Rarα in adrenocortical development. (**A**) Rarα regulates the expression of genes involved in the regulation of Wnt non-canonical pathway (*Wnt4*, *Tcf3*, *Lef1…*), angiogenesis (*Vegfa*) and Extra Cellular Matrix integrity (*Fibronectin1*, *Collagen 3α1*…) contributing to the organization of the adrenal cortex. Wnt4 activated pathway contributes to the differentiation of ZG cells, Vegfa to normal angiogenesis and Fibronectin1 and Collagen 3α1 being components of the Extra Cellular Matrix. (**B**) Model in which a homeostatic equilibrium between Rarα, Wnt and Vegf signaling pathways is required for the normal development of the vasculature and Extra Cellular Matrix structure, leading to normal adrenal cortex organization.

In summary, our study identifies RARα as contributing to the maintenance of normal adrenal cortex structure and cell proliferation by modulating Wnt signaling. The absence of adrenal nodules in 52 weeks old mice suggests that abnormal cell proliferation induced by the absence of RARα signaling is not enough to promote APA, but may constitute one of the elements involved in the pathogenesis of PA. We propose a model in which a homeostatic equilibrium between retinoic acid, Wnt and Vegf signaling pathways is required for the maintenance of normal adrenal cortex structure in a specific time-dependent manner that differs between males and females (Fig. 7). In addition to previously reported regulators and to further elements that remain to be identified, Ddysregulation of this equilibrium in adult adrenals may contribute to abnormal cell proliferation, creating the propitious environment for the emergence of specific driver mutations in PA.

## Methods

### Patients

Patients with primary aldosteronism were recruited between 2002 and 2006 through the COMETE (COrtico- et MEdullo-surrénale: les Tumeurs Endocrines) network (COMETE-HEGP protocol), approved by the French Research ethics committee (Comité de Protection des Personnes, CPP) under authorization number CPP 2012-A00508-35. Methods for screening and subtype identification of PA were performed according to institutional and Endocrine Society guidelines^78,79^. In patients diagnosed with primary aldosteronism, a thin slice CT scan or MRI of the adrenal and/or an adrenal venous sampling were performed to differentiate between unilateral and bilateral form. All patients gave written informed consent for genetic and clinical investigation.

### Microarray analysis on human tissues

Transcriptome analyses were performed on 48 APA samples and eleven control adrenals obtained from enlarged nephrectomies for renal carcinoma. Total RNA was isolated from frozen tissue using Trizol (Invitrogen) and then cleaned-up on silica columns using RNeasy Mini Kit (Qiagen). The integrity and quality of the RNA were systematically checked using an Agilent 2100 Bioanalyzer with the RNA6000 Nano Assay (Agilent Technologies). For APA samples, complementary RNA was synthesized from 15 μg of total RNA using the Chemiluminescent RT Labeling Kit (Applied Biosystems). For control adrenals, complementary cRNA was synthesized from 500 ng of total RNA using the NanoAmpTM RT-IVT Labeling Kit (Applied Biosystems). Samples were hybridized to microarrays using Applied Biosystems chemiluminescence detection kit according to the manufacturer’s protocol. Acquisition of the chemiluminescence images and primary data analysis were done using Applied Biosystems Expression Array System Software v1.1.1. Microarray gene expression data from the primary analysis for each sample was median centered, log 2 transformed, model-adjusted, and quality controlled^80^. Mean expression profiles for each biological condition were obtained by variance-weighted averaging normalized expression values. Pairwised inter-array normalizations were carried out using the NeONORM method described previously^81^. Fold change values were expressed on log2 scale. P-values were determined based on a normal distribution hypothesis of signal intensities using standard methods^82^. Multiple probes for a single gene, cross-reactivity of a single probe to several genes, as well as the resolution of probe-ID annotations were done as defined previously^83^. Hierarchical clustering based on Euclidean distance and complete-linkage was carried out using the R-packages *hclust* and *gplot*. Differentially expressed genes among groups were calculated using a fold-change z-test with positive false discovery rate correction. P values for over- and under-representation of pathways were calculated using a binominal distribution and a Bonferoni correction for multiple testing.

The data for the human APA transcriptome analysis have been deposited to the public data repository http://mace.ihes.fr under accession nos.: 2575793518 (controls) and 2293267822 (APA).

### Mice

Animal studies were conducted according to the guidelines and regulations formulated by the European Commission for experimental use (Directive 2010/63/EU) and were approved by the local Ethics committee of Paris Descartes University (N°17-020) and by the French Ministère de l′Enseignement Supérieur, de la Recherche et de l′Innovation (APAFIS authorization number #11509-201703281427107 v4). *Rarα*^−/−^ mice (constitutive homozygous deletion of exon 8 of *Rarα* gene, leading to inactivation of the protein) have been kindly provided by Dr Norbert Ghyselinck and Dr Pierre Chambon^32^. They were maintained and bred on a mixed background and littermates control animals were used in all experiments. All experiments were conducted on male and female mice between 12 and 52 weeks of age. Blood were collected in abdominal aorta in lithium-heparin blood collection tubes (Microvette CB300LH, Sarstedt). The right adrenals were frozen in liquid nitrogen and the left adrenals fixed in 4% paraformaldehyde.

### Magnetic resonance imaging

Anesthesia was induced with 4% isoflurane and maintained at a 1% level during imaging. Magnetic resonance imaging was performed with a dedicated small-animal 4.7 Tesla MR system (Biospec 47/40 USR Brucker). The animals were placed in a supine position on a four-channel phased array receive coil inside a volume transmit coil (BruckerBiospin, Ettlingen, Germany). We performed two anatomic sequences (TurboRARA T2 and FISP) in two directions (coronal and sagittal) to optimized measurement of the size of the adrenal. The thickness of the slices was 0.7 mm and the resolution was 175×175 μm and 156×126 μm respectively.

### Aldosterone, corticosterone and plasma renin concentration measurement in murine plasma samples

For steroids quantification, 150 μl of heparinized murine plasma was skiped with 500 ng of stable isotope labeled internal standards for aldosterone and corticosterone. Following C18-based solid-phase-extraction, samples were subjected to LC-MS/MS analysis using a reversed-phase analytical column (Acquity UPLC® C18, Waters, Milford, MA, USA) operating in line with a XEVO TQ-S triple quadrupole mass spectrometer (Waters Xevo TQ/S, Milford, MA, USA) in multiple reaction monitoring mode. Internal standards were used to correct for analyte recovery across the sample preparation procedure in each sample. Analyte concentrations were calculated from integrated chromatograms considering the corresponding responses determined in appropriate calibration curves in plasma matrix.

The plasma renin concentration was determined in murine plasma samples by determining the Ang I formation rate in the presence of excess of angiotensinogen, where it becomes solely dependent on the concentration of active renin. Therefore, diluted plasma was supplemented with an excess of recombinant murine angiotensinogen. Samples were incubated at 37°C in the presence of an Ang I stabilizing inhibitor cocktail for 60 minutes at 37°C. Following LC-MS/MS based quantification of Ang I as described^84^, the Ang I formation rate was calculated by subtracting Ang I values obtained in non-incubated control samples and expressed in [(ng Ang I/ml)/h].

### RNA extraction and RT-qPCR analysis

Total RNA was extracted using Janke and Kunkel’s Ultra-Turrax T25 (IKA technologies, Staufen DE) in Trizol reagent (Ambion Life Technologies, Carlsbad CA) according to the manufacturer’s recommendations. After deoxyribonuclease I treatment (Life Technologies, Calsbad CA), 500 ng of total RNA were retro-transcribed (iScript reverse transcriptase, Biorad, Hercules, CA). The quantitative qPCR was performed using SYBRgreen (Sso advanced universal SyBr Green Supermix, Biorad, Hercules, CA) on a C1000 touch thermal cycler of Biorad (CFX96 Real Time System) according to the manufacturer’s instructions. Controls without template were included to verify that fluorescence was not overestimated from primer dimer formation or PCR contaminations. RT-qPCR products were analyzed in a post amplification fusion curve to ensure that a single amplicon was obtained. Normalization for RNA quantity and reverse transcriptase efficiency was performed against three reference genes (geometric mean of the expression of Ribosomal 18S RNA, β2-microglobulin and Ubiquitin C for mouse samples and 18S RNA, GAPDH and HPRT for human samples), in accordance with the MIQE guidelines^85^. Quantification was performed using the standard curve method. Standard curves were generated using serial dilutions from a cDNA pool of all samples. The following primers were used: 5′-CCCTGCCCTTTGTACACACC-3′ and 5′-CGATCCGAGGGCCTCACTA-3′ for *18S*; 5′-ATTCACCCCCACTGAGACTG-3′ and 5′-TGCTATTTCTTTCTGCGTGC-3′ for *mβ2-microglobulin*; 5′-AGCCCAGTGTTACCACCAAG-3′ and 5′-ACCCAAGAACAAGCACAAG-3′ for *mUbiquitinC*; 5′-GTGCTTCATCCACTGGCTGGAA-3′ and 5′-GTCTGCGATAGGACCTGGTTGA-3′ for *mStAR*; 5′-TGCTCAACCTGCCTCCAGACTT-3′ and 5′-ACTGGCTGAAGTCTCGCTTCTG-3′ for *mCyp11a1*; 5′-TGTATCGAGAGCTGGCAGAG-3′ and 5′-CCTGGATGGCATCCATTGAC-3′ for *mCyp11b1*; 5′-ACCTACAGTGGCATTGTG-3′ and 5′-GATTGCTGTCGTGTCAAC-3′ for *mCyp11b2*; 5′-CCCTGTCTTTGGGAAGGTGGTG-3′ and 5′-CACCTGCTGAAGAGATGGCGTATAC-3′ for *mWnt4*; 5′-CAGATGGTGGCCTGGATACT-3′ and 5′-CATCCCTGCTGTAGCTGTCA-3′ for *mTcf3*; 5′-AAATGGGTCCCTTTCTCCAC-3′ and 5′-TCGTCGCTGTAGGTGATGAG-3′ for mLef1; 5′-AGCCTAAAGGTCTTATGTGG-3′ and 5′-ATGGAATCGTCGGTCAGT-3′ for *mAxin2*; 5′-CCTGGTGGACATCTTCCAGGAGTACC-3′ and 5′-GAAGCTCATCTCTCCTATGTGCTGGC-3′ for *mVegfa*; 5′-GGGAAGAAGTTCCACCATCA-3′ and 5′-ATGTGGCCTTTTCCAATACG-3′ for *mVegfc*; 5′-TCAAGTCAGCAACGTGGAAG-3′ and 5′-TATCGAGGCTGTGTCGACTG-3′ for *mHif1α*; 5′-CGGGAATGCGAGCTCTATGT-3′ and 5′-CTCGAGTCAGTACGGATGCC-3′ for *mPrkar1a*; 5′-CCCGAGATTATCCTGAGCAA-3′ and 5′-ATAGGCTGGTCAGCGAAGAA-3′ for *mPrkaca*; 5′-TCCACAAATACTCAGGCAAAGAG-3′ and 5′-GCAGCTCCCTGGTCAGTAG-3′ for *mS100a4*; 5′-GTCTCCTCTGACTTCAACAGCG-3′ and 5′-ACCACCCTGTTGCTGTAGCCAA-3′ for *hGAPDH*; 5′-CTCAACTTTAACTGGAAAGAATGTC-3′ and 5′-TCCTTTTCACCAGCAAGCT-3′ for *hHPRT*; 5′-GACCAGATCACCCTCCTCAA-3′ and 5′-GTCCGAGAAGGTCATGGTGT-3′ for *hRARα*.

### Immunostainings

Immunohistochemistry and immunofluorescence were performed on Formalin-Fixed Paraffin-Embedded mouse adrenals from 12 and 52 week-old WT and KO male mice. Adrenal sections were deparaffinized in xylene and rehydrated through graded ethanol. To unmask the antigen, the slides were incubated in antigen unmasking solution (Vector laboratories Ltd) for 30 minutes at 98°C.

Immunofluorescence was performed for Dab2 (1/100, sc-13982, Santa Cruz Biotechnology), Laminin β1 (1/500, ab69633, Abcam), β-catenin (1/1000, 61054, BD Transduction Laboratories), p-Creb (1/800, 9198, Cell Signaling Technology), mouse Cyp11b2 (mCyp11b2, 1/100, generous gift from CE Gomez-Sanchez), mouse Cyp11b1 (mCyp11b1, 1/50, generous gift from CE Gomez-Sanchez) and mouse podocalyxin (1/100, AF1556, R&D Systems). Tissues were permeabilized by a treatment of 15 min with TBS 0.1% Triton X100. The sections were then blocked with 5% BSA diluted in TBS for Dab2, laminin β1, β-catenin and p-Creb or with 10% normal donkey serum diluted in TBS 0.1% Triton X100 for mCyp11b2 and mCyp11b1. Primary antibodies were diluted in 3% BSA diluted in TBS (for Dab2, laminin β1, β-catenin and p-Creb) or in 5% normal donkey serum and 10% normal rat serum diluted in TBS 0.1% Triton X100 (for mCyp11b2 and mCyp11b1) and incubated overnight at 4°C (for laminin β1, β-catenin, p-Creb, mCyp11b2 and mCyp11b1) or 1 h at room temperature (Dab2). Primary antibodies were detected with donkey anti-rabbit Alexa 594 (1/400 for Dab2, laminin β1 and p-Creb, 1/500 for mCyp11b2, A21207, Thermo Fisher), donkey anti-mouse Alexa 594 (1/400 for β-catenin, A21203, Thermo Fisher), Donkey anti-goat Alexa 594 (1/400 for mouse podocalyxin, A11058, Thermo Fisher) and Donkey anti-sheep Alexa 488 (1/500 for mCyp11b1, A11015, Thermo Fisher). Nuclei were conterstained using 4′,6-diamidino-2-phenylindole (DAPI) (1/5000, Roche Diagnostics GmbH).

Immunohistochemistry was performed for mouse Ki67 (1/200, RM-9106, Thermo Fisher), mouse 20-αHSD (1/5000, generous gift from Y Weinstein), human RARα (1/500, ab28767, Abcam) and human CYP11B2 (hCYP11B2, 1/100, generous gift from CE Gomez-Sanchez). Sections were incubated in 3% hydrogen peroxide (Sigma-Aldrich) for 10 min to inhibit endogenous peroxidases. Non-specific staining was blocked by incubating sections 30 min with 10% normal goat serum diluted in PBS for Ki67, 20-αHSD and RARα or 10% horse serum (HS) and 0.5% SDS diluted in Tris 0.1 M pH 7.4 for hCYP11B2. Primary antibodies were incubated overnight at 4°C: Ki67, 20-αHSD and RARα were diluted in 1% NGS or HS in PBS and CYP11B2 was diluted in 10% horse serum and 0.2% Tween 20 in Tris 0.1 M pH 7.4. Primary antibodies were detected by incubation for 30 min with affinity-purified goat anti-rabbit (1/400 for Ki67 and 20-αHSD; Vector Laboratories) or rabbit anti-goat (1/400 for RARα; Vector Laboratories) diluted in 1% normal goat serum in PBS or horse anti mouse (1/400 for hCYP11B2; Vector Laboratories) diluted in 10% horse serum and 0.2% Tween 20 in Tris 0.1 M pH 7.4. After incubation for 30 min with an avidin-biotin-peroxidase complex (Vectastain ABC Elite; Vector Laboratories), the slides were developed using diaminobenzidin (Vector Laboratories) and counter-stained using Hematoxylin Harris Heamatoxylin, RAL Diagnostics).

All microscopic examinations were done with a x10 and x20 objectives lens using Leica confocal or Vectra^TM^ system.

Multispectral images were acquired using a Vectra® automated imaging system and automatically quantified with InForm® software (both Perkin Elmer). Results of quantification are expressed in percentage of Dab2 or Ki67 positive cells over total adrenal cortex cells. Statistical significance was assessed among groups using *t* test or Mann-Whitney.

### Histological examination

Histological examination was performed on 6 μm sections stained with hematoxylin-eosin safran (HES) and with Sirius red. Microscopic examinations were done with a x10 and x20 objectives lens using Vectra^TM^ system or with a x40 objective lens using confocal SP8 Leica microscope.

### Western blot

Western Blot analysis was performed on mouse adrenals from 12 and 52 week-old WT and KO male mice. Total proteins were extracted using RIPA buffer (RB 4475, BioBasic Canada) with EDTA-free protease and phosphatase inhibitor (Bimake), proteins were then spun on a rotor for 30 min and centrifuged at 13000 rpm for 15 min, the proteins were then recovered and concentration determined using Bradford protein essay (Biorad). 10 µg of total proteins were loaded on 10% Acrylamide SDS-PAGE gel, transferred onto nitrocellulose membrane and tagged with the following antibodies: phospho-β-Catenin (Thr41/Ser45; 1/1000, 9565, Cell Signaling Technology), phospho-β-Catenin (Ser552; 1/1000, 5651, Cell Signaling Technology), phospho-β-Catenin (Ser675; 1/1000, 4176, Cell Signaling Technology), β-Catenin (1/1000, 8480, Cell Signaling Technology), phospho-CREB (Ser133; 1/1000, 9198, Cell Signaling Technology), CREB (1/1000, 9197, Cell Signaling Technology)anti-rabbit IgG, HRP-linked antibody (1/2000, 7074, Cell Signaling technology), β-actin (1/5000, 2228, Sigma-Aldrich) and anti-mouse IgG, HRP-linked antibody (1/5000, A90-146P, Bethyl Laboratories). The signals were developed by Clarity Max™ Western ECL substrate (Biorad, Hercules, CA) and detected by Fujifilm Las-4000 mini Luminescent image analyzer (Fujifilm, Tokyo-Japan) and quantified by Multi gauge software (Fujifilm, Tokyo-Japan). Expression of the phosphoprotein was normalized to the expression of the corresponding total protein and the expression of total proteins was normalized to the expression of the housekeeping protein β-actin.

### Microarray analysis of mouse adrenal gene expression

Adrenal gene expression profiles for 16 mice (4 *Rarα*^+/+^ and 4 *Rarα*^−/−^ 12 weeks old mice and 4 *Rarα*^+/+^ and 4 *Rarα*^−/−^ 52 weeks old mice) were analyzed using Affymetrix Clariom S Mouse array. After validation of the RNA quality with Bioanalyzer 2100 (using Agilent RNA6000 nano chip kit), 100 ng of total RNA was reverse transcribed following the GeneChip® WT Plus Reagent Kit (Affymetrix). Briefly, the resulting double strand cDNA was used for *in vitro* transcription with T7 RNA polymerase (all these steps are included in the WT cDNA synthesis and amplification kit of Affymetrix). After purification according to Affymetrix protocol, 5.5 μg of Sens Target DNA were fragmented and biotin labeled. After control of fragmentation using Bioanalyzer 2100, cDNA was then hybridized to GeneChip® Clariom S Mouse (Affymetrix) at 45°C for 17 hours. After overnight hybridization, chips were washed on the fluidic station FS450 following specific protocols (Affymetrix) and scanned using the GCS3000 7G. The scanned images were then analyzed with Expression Console software (Affymetrix) to obtain raw data (cel files) and metrix for Quality Controls.

Analyses were performed using TAC (Transcriptome Analysis Console) software. Genes with adjusted P value <0.05 and log2 fold changes <−1 or >1 were considered to be down- or up-regulated.

Molecular function and biological process enrichment analyses were performed using Gene Ontology (www.geneontology.org). For the analyses, we used the list of differentially expressed genes in 12 or 52 weeks old *Rarα*^+/+^ and *Rarα*^−/−^ mice. The analysis criteria were kept as default values. The data discussed in this publication have been deposited in NCBI's Gene Expression Omnibus and are accessible through GEO Series accesion number GSE136801 (https://www.ncbi.nlm.nih.gov/geo/query/acc.cgi?acc=GSE136801).

### Statistics

Data are shown in mean ± SEM. Statistical significance was assessed between the groups using unpaired t-test or Mann-Whitney test (Prism, GraphPad Software, USA). A *p*-value <0.05 was considered statistically significant.

## Supplementary information

Online Supplementary Information

Supplementary Table 1

Supplementary Table 2

Supplementary Table 3

Supplementary Table 4

Supplementary Table 5

Supplementary Table 6

Supplementary Table 7

Supplementary Table 8

Supplementary Table 9

Supplementary Table 10

Supplementary Table 11

Supplementary Table 12

Supplementary Table 13
