# Supplementary material for: Retinoic acid receptor α as a novel contributor to adrenal cortex structure and function through interactions with Wnt and Vegfa signalling
Source: Sci Rep. 2019 Oct 11;9:14677. doi: 10.1038/s41598-019-50988-2 (PMC6789122; doi:10.1038/s41598-019-50988-2)
Supplement: Supplementary file 1 — Online Supplementary Information [file 41598_2019_50988_MOESM1_ESM.pdf]

Online Supplementary Information

Retinoic acid receptor α as a novel contributor to adrenal cortex structure and function through interactions with Wnt and Vegfa signalling

Rami M El Zein[1,2], Audrey H Soria[1,2], Jose Felipe Golib Dzib[3], Amanda J. Rickard[1,2], Fabio L Fernandes-Rosa[1,2], Benoit Samson-Couterie[1,2], Isabelle Giscos-Douriez[1,2], Angélique Rocha[1,2], Marko Poglitsch[4], Celso E Gomez-Sanchez[5], Laurence Amar[1,2,6], Norbert B Ghyselinck[7], Arndt Benecke[3,8], Maria-Christina Zennaro[1,2,9*$], Sheerazed Boulkroun[1,2*$]

[1]INSERM, UMRS_970, Paris Cardiovascular Research Center, Paris, France
[2]Université Paris Descartes, Sorbonne Paris Cité, Paris, France
[3]Centre National de la Recherche Scientifique (CNRS), Institut des Hautes Etudes Scientifiques, Bures sur Yvette, France
[4]Attoquant Diagnostics, Vienna, Austria
[5]Division of Endocrinology, G.V. (Sonny) Montgomery VA Medical Center and University of Mississippi Medical Center, Jackson, MS 39216, USA
[6]Assistance Publique-Hôpitaux de Paris, Hôpital Européen Georges Pompidou, Unité Hypertension artérielle, Paris, France
[7]Institut de Génétique et de Biologie Moléculaire et Cellulaire (IGBMC), Département de Génétique Fonctionnelle et Cancer; CNRS, UMR7104, Illkirch, France ; INSERM U1258, Illkirch, France; Université de Strasbourg (UNISTRA), Illkirch Cedex, France
[8]Present address: CNRS UMR8246, NPS, Sorbone University, Paris, France
[9]Assistance Publique-Hôpitaux de Paris, Hôpital Européen Georges Pompidou, Service de Génétique, Paris, France

[$] Equal contribution
[*] Corresponding author

Address correspondence to:
Sheerazed Boulkroun, PhD
INSERM, U970
Paris Cardiovascular Research Center – PARCC
56, rue Leblanc,
75015 Paris – France
Tel : +33 (0)1 53 98 80 24
Fax : + 33 (0)1 53 98 79 52
e-mail : sheerazed.boulkroun@inserm.fr

Maria-Christina Zennaro, MD, PhD
INSERM, U970
Paris Cardiovascular Research Center – PARCC
56, rue Leblanc,
75015 Paris – France
Tel : +33 (0)1 53 98 80 42
Fax : + 33 (0)1 53 98 79 52
e-mail : maria-christina.zennaro@inserm.fr

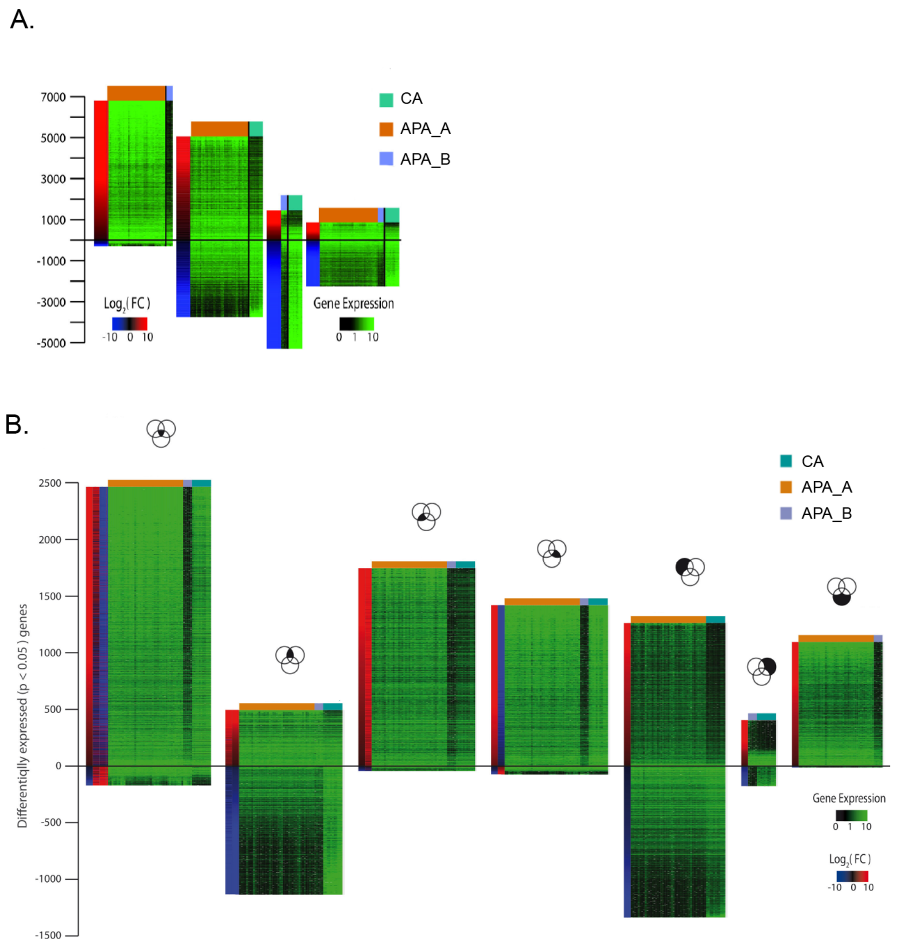

Supplementary Figure 1: Heat-maps of the seven sets of differentially expressed genes from pairwise comparisons among two subpopulations of APA and controls. (A) Heat-maps of the statistically significantly (post-hoc P<0.01) differentially expressed genes between control adrenals (cyan), APA_A (orange) and APA_B (purple). (B) Heat-maps of the statistically significantly (post-hoc p<0.05) differentially expressed genes between the seven groups of samples identified. Samples separated into three different groups are depicted as control adrenals (CA), APA Group A (APA_A) and APA Group B. The seven sets of overlapping and paired-wise-comparison-specific genes are delineated at the bottom. (C) Expression of *RARα* was investigated by RT-qPCR on mRNA extracted from 6 control adrenals and 11 APA. Values are presented as the mean ± SEM; p values were calculated using a two-sided Mann-Whitney test. *, p<0.05.

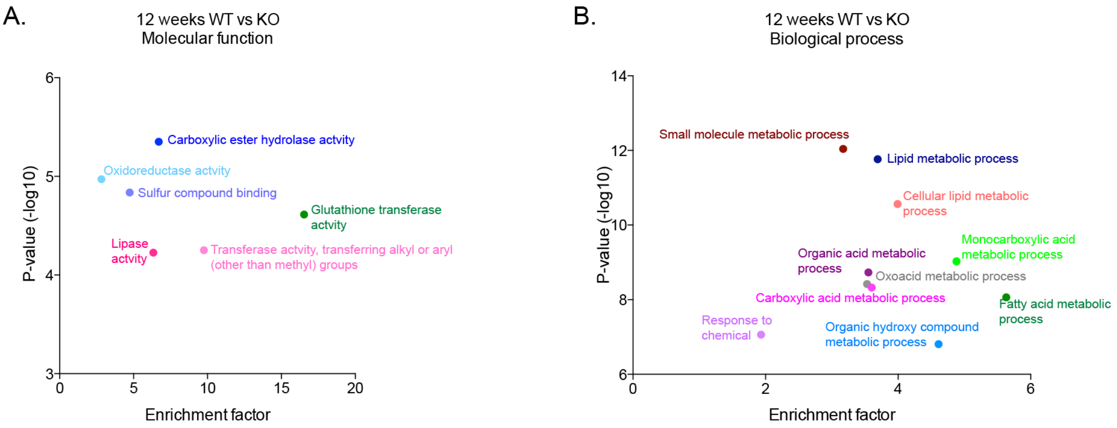

Supplementary Figure 2: Molecular function and biological process enrichment analyses in *Rarα*$^{-/-}$ mice compared to *Rarα*$^{+/+}$. Molecular function and biological process enrichment analyses were performed using Gene Ontology. (A) Molecular function and (B) biological process enrichments were determined using the list of differentially expressed genes in adrenal from 12 weeks old *Rarα*$^{+/+}$ and *Rarα*$^{-/-}$ mice.

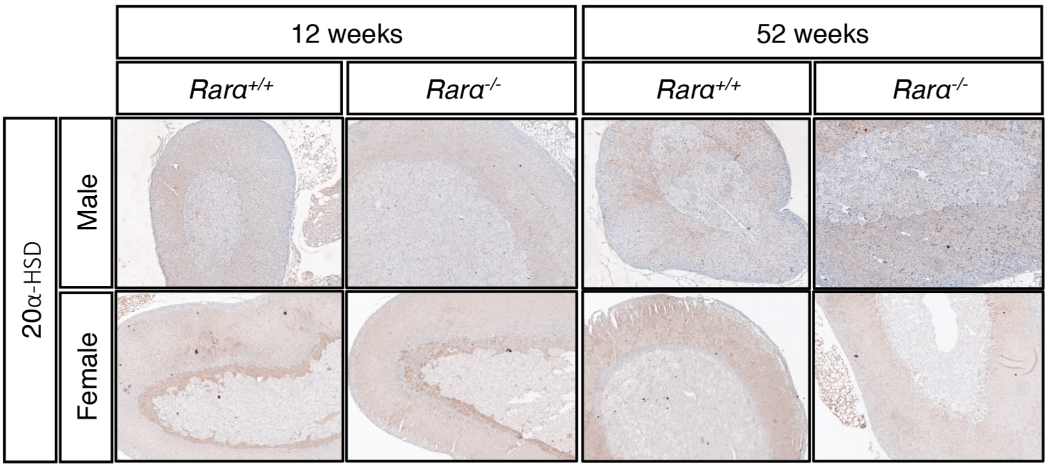

Supplementary Figure 3: *Rarα* does not affect X-zone. 20-αHSD expression in adrenals from male and female 12 and 52 weeks old *Rarα*$^{+/+}$ and *Rarα*$^{-/-}$ mice detected by immunohistochemistry.

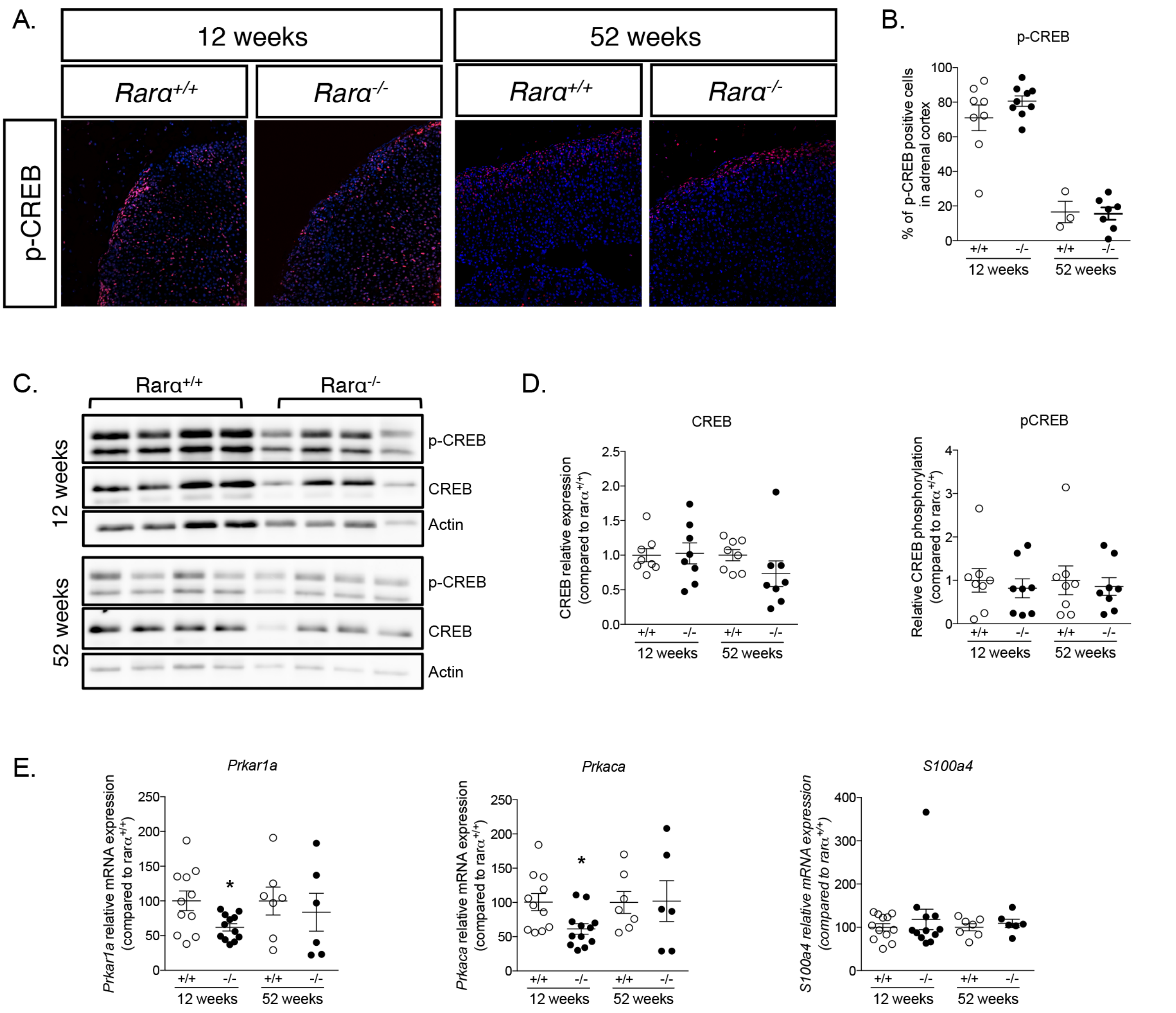

Supplementary Figure 4: *Rara* does not affect PKA signaling pathway in male mice. (A) p-CREB expression in adrenals from 12 and 52 weeks old *Rarα*$^{+/+}$ and *Rarα*$^{-/-}$ mice detected by immunofluorescence. (B) Percentage of p-CREB positive cells was quantified using an automated imaging platform. (C) Expression of CREB and p-CREB in response to *Rarα* invalidation in adrenal was evaluated by western blot in eight *Rarα*$^{+/+}$ and *Rarα*$^{-/-}$ adrenal from 12 and 52 weeks old mice. (D) Quantification of CREB and p-CREB expression detected by western blot. (E) The expression of *Prkar1a*, *Prkaca* and *S100a4* was investigated by RT-qPCR. Values are presented as mean ± SEM; p values were calculated using a Mann-Whitney test or *t*-test. *, p<0.05.

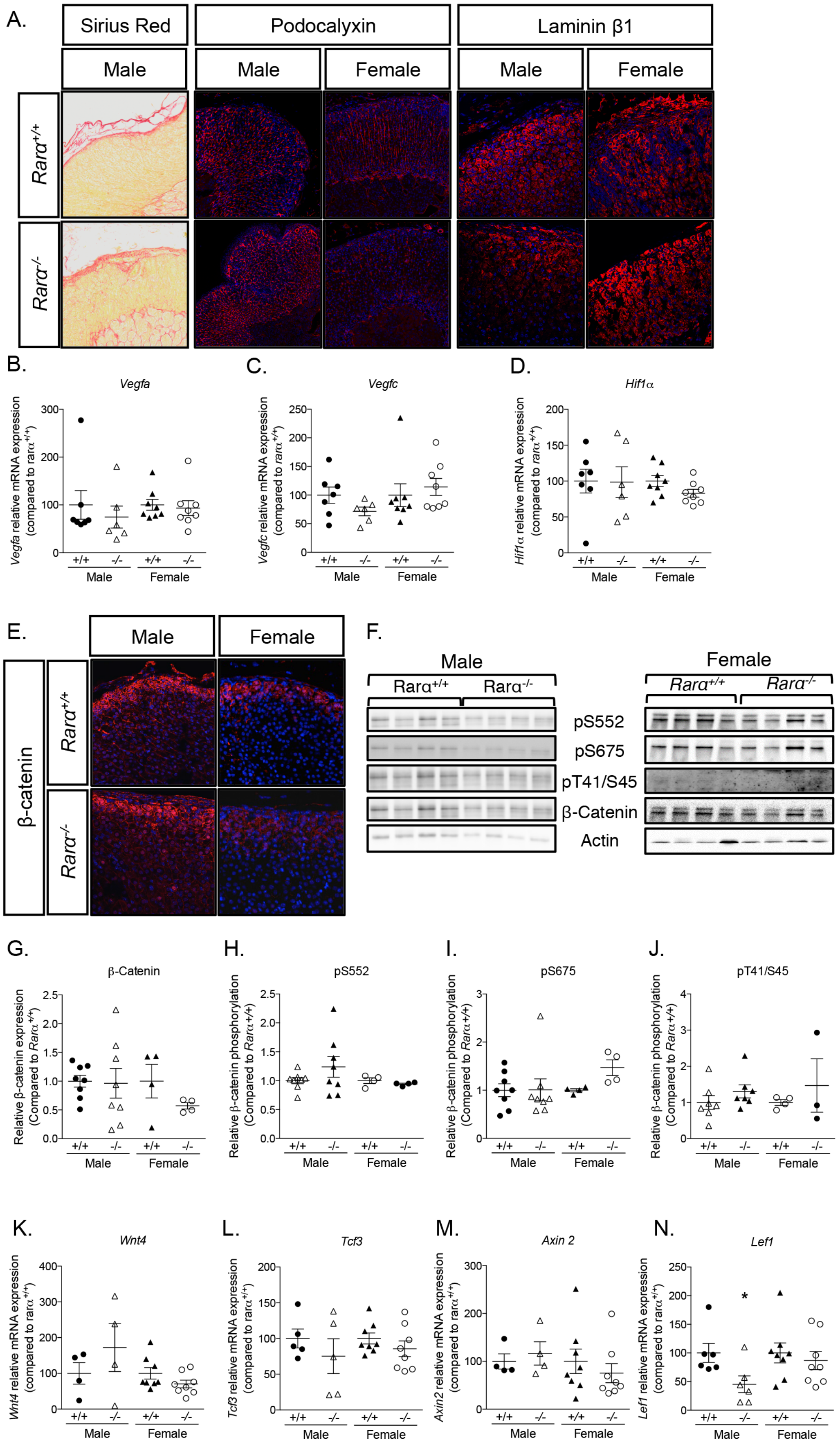

A.
Sirius Red
Podocalyxin
Laminin β1
Male
Male
Female
Male
Female
Raraα+/+
Raraα-/-
B.
Vegfa
Vegfa relative mRNA expression (compared to rarα+/+)
C.
Vegfc
Vegfc relative mRNA expression (compared to rarα+/+)
D.
Hif1α
Hif1α relative mRNA expression (compared to rarα+/+)
+/+ -/- +/+ -/-
Male Female
E.
Male
Female
β-catenin
Raraα+/+
Raraα-/-
F.
Male
Female
Rarα+/+
Rarα-/-
pS552
pS675
pT41/S45
β-Catenin
Actin
G.
β-Catenin
Relative β-catenin expression (Compared to Rarα+/+)
H.
pS552
Relative β-catenin phosphorylation (Compared to Rarα+/+)
I.
pS675
J.
pT41/S45
K.
Wnt4
Wnt4 relative mRNA expression (compared to rarα+/+)
L.
Tcf3
Tcf3 relative mRNA expression (compared to rarα+/+)
M.
Axin 2
Axin2 relative mRNA expression (compared to rarα+/+)
N.
Lef1
Lef1 relative mRNA expression (compared to rarα+/+)
*

Supplementary Figure 5: *Rarα* inactivation does not alter Wnt/β-catenin pathway in 52 weeks old male and female mice. (A) Expression of β-catenin was evaluated by immunofluorescence in 12 weeks old male and female *Rarα*$^{+/+}$ and *Rarα*$^{-/-}$ mice. (B) Expression and phosphorylation of β-catenin in response to *Rarα* invalidation. Proteins were extracted form total adrenal and submitted to western blot analysis. Phosphorylation/dephosphorylation in activating (pS552 and pS675) and inactivating (pT41/S45) residues and total expression of β-catenin was investigated. (C-F) Quantification of β-catenin expression (C) and of phospho-specific signals in inactivating (D) and activating (E, F) residues was performed in *Rarα*$^{+/+}$ and *Rarα*$^{-/-}$ adrenal. (G-J) The expression of *Wnt4* (G), *Tcf3* (H), *Lef1* (I) and *Axin2* (J) was investigated by RT-qPCR on mRNA extracted from 4 to 8 adrenals from *Rarα*$^{+/+}$ and *Rarα*$^{-/-}$ male and female mice. Values are presented as mean ± SEM; p values were calculated using a Mann-Whitney test or *t*-test. *, $p<0.05$

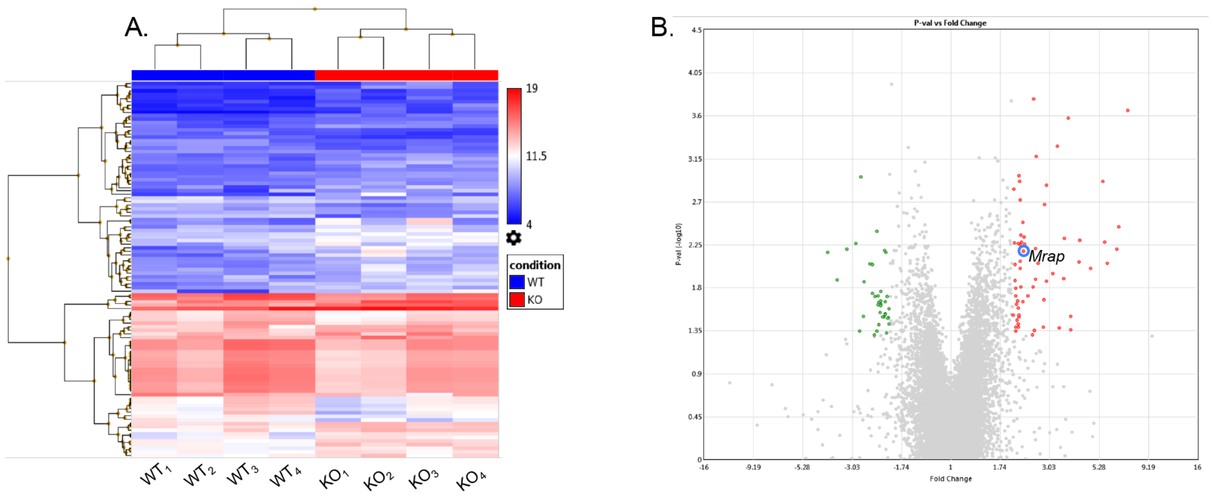

Supplemental Figure 6: *Rarα* modifies adrenal transcriptome profile at 52 weeks of age. (A) Hierarchical clustering of samples using the 243 genes differentially expressed genes in adrenals from 52 weeks old *Rarα*$^{+/+}$ and *Rarα*$^{-/-}$ male animals (4 animals per group). (B) Volcano plot showing the differentially expressed genes in 52 weeks old *Rarα*$^{+/+}$ and *Rarα*$^{-/-}$ animals. The x-axis is the fold change between the two conditions; the adjusted p value based on $-\log_{10}$ is reported on the y-axis. Genes significantly different are highlighted as green (down-regulated in *Rarα*$^{-/-}$ mice) or red (up-regulated in *Rarα*$^{-/-}$ mice) dots.
